# Transcriptomics Responses in Marine Diatom *Thalassiosira pseudonana* Exposed to the Polycyclic Aromatic Hydrocarbon Benzo[a]pyrene

**DOI:** 10.1371/journal.pone.0026985

**Published:** 2011-11-03

**Authors:** Raquel N. Carvalho, Stephanie K. Bopp, Teresa Lettieri

**Affiliations:** Rural, Water, and Ecosystem Resources Unit, Institute for Environment and Sustainability, European Commission - Joint Research Centre, Ispra, Varese, Italy; UCLA-DOE Institute for Genomics and Proteomics, United States of America

## Abstract

Diatoms are unicellular, photosynthetic, eukaryotic algae with a ubiquitous distribution in water environments and they play an important role in the carbon cycle. Molecular or morphological changes in these species under ecological stress conditions are expected to serve as early indicators of toxicity and can point to a global impact on the entire ecosystem. *Thalassiosira pseudonana*, a marine diatom and the first with a fully sequenced genome has been selected as an aquatic model organism for ecotoxicological studies using molecular tools. A customized DNA microarray containing probes for the available gene sequences has been developed and tested to analyze the effects of a common pollutant, benzo(a)pyrene (BaP), at a sub-lethal concentration. This approach in diatoms has helped to elucidate pathway/metabolic processes involved in the mode of action of this pollutant, including lipid metabolism, silicon metabolism and stress response. A dose-response of BaP on diatoms has been made and the effect of this compound on the expression of selected genes was assessed by quantitative real time-PCR. Up-regulation of the long-chain acyl-CoA synthetase and the anti-apoptotic transmembrane Bax inhibitor, as well as down-regulation of silicon transporter 1 and a heat shock factor was confirmed at lower concentrations of BaP, but not the heat-shock protein 20. The study has allowed the identification of molecular biomarkers to BaP to be later on integrated into environmental monitoring for water quality assessment.

## Introduction

In the last decade toxicogenomics has been widely applied to the ecotoxicology field. DNA microarray of aquatic species enable a top-down approach in the identification of molecular endpoints associated with environmental stressors, for review see [1], [2], [3]. DNA microarrays have become mostly a routine approach in many laboratories ever since the price dropped down, thus facilitating the investigation on the mode of action of several chemical pollutants as well as other environmental stressors. In the area of environmental biomonitoring, this methodology has some advantages, since it is able to decipher chemical mode of action of chemicals, including mixtures and emerging pollutants for which there is scarce information on the affected pathways in organisms.

Recently, the need for reliable bioassays as complementary methods for the detection of pollutants has been acknowledged, to allow the fast screening of samples and serve as an early warning system [4]. Current focus is on the development of screening tools to assess the ecosystem quality based on the integration of several endpoints at the molecular, cellular and population levels [5]. Aquatic ecotoxicogenomics studies, although they have been more focused on model and non-model fish species [6], [7], [8], [9], in the last years have also included other ecological relevant organisms [10].

Diatoms are important organisms because of their ubiquitous distribution in aquatic environments and their unmatched role in the global carbon fixation. They are believed to account for about 40% of the total carbon fixation in oceans and 25% of world carbon fixation, closely matching the contribution of the world rain forests [11]. Most of the organic carbon produced by diatoms in oceans is rapidly consumed and sustains marine food webs. When xenobiotics are present in the environment, diatoms may facilitate the uptake of contaminants into higher organisms, increasing the bioaccumulation and possibly the toxicity. For this reason, the detection of pollutants in diatoms or their effects at the molecular level may serve as a diagnostic tool of water quality, facilitating early environmental risk assessments.

Furthermore, increased knowledge of diatoms metabolic pathways will facilitate their use in promising new biotechnological applications. These include the use of diatoms as an alternative source for biofuel production in detriment of land plants [12] as well as the use of the nano-biosilica casing of diatoms for drug delivery in nano-medical applications [13].

The full genome sequence and annotation of the centric diatom *Thalassiosira pseudonana* [14] and the pennate diatom *Phaeodactylum tricornutum* [15] are available and the sequencing of additional diatom species has been either completed recently or is undergoing (DOE Joint Genome Institute: http://www.jgi.doe.gov/genome-projects/). Furthermore, subsequent molecular studies on diatoms have greatly increased the available information on conserved eukaryotic pathways as well as specific pathways of these organisms, in particular their molecular response mechanisms to stress conditions or the silicon metabolic pathway. Diatoms, as primary producers in aquatic environments, represent promising biological tools for the discovery of mode of action of environmental stressors, in a time frame compatible with a rapid screening of water samples.

We report here the development of a customized microarray containing the gene pool of the diatom *Thalassiosira pseudonana*. This microarray has been used to investigate the effects of exposure to a common pollutant in aquatic environments at the molecular level.

In our first DNA Microarray studies, we analyzed the effect of benzo[a]pyrene (BaP), a polycyclic aromatic hydrocarbon (PAH) compound. PAHs are common trace pollutants in freshwater and marine sediments worldwide that pose major threats to aquatic life, particularly near areas of intense anthropogenic activity, and they may derive from different sources including fossil fuels and burning of organic matter [16]. Several PAHs are included in the priority substance list under the EC Water Framework Directive [17]. BaP is one of these priority substances and has been linked with mutagenic and carcinogenic effects among others in exposed organisms [18].

The current study aims at the identification of the mode of action of BaP in diatoms, which are expected to be one of the most relevant entry points of contaminants in marine food webs. Since diatoms are unique eukaryotic organisms sharing many features to both animals and plants [14], they are particularly suitable for the development of monitoring biomarkers that are transferable across species.

## Results

### Microarray Gene Expression Analysis

Microarray experiments were performed to compare the gene expression profile of BaP-exposed diatom cultures with the untreated controls. By using a sub-lethal dose of pollutant inducing about 40% growth inhibition and a 24 h exposure time there was still a significant response at the molecular level triggered by BaP exposure. After statistical analysis and correction of the DNA microarray data (see Materials and Methods), 653 genes with regulation ≥2 fold were identified (Table S1), which constitutes about 6% of the total genes on the array. The highest fold regulation was observed for a small heat-shock protein (ID 261072), with 147 fold increase in gene expression.

**Figure 1:**
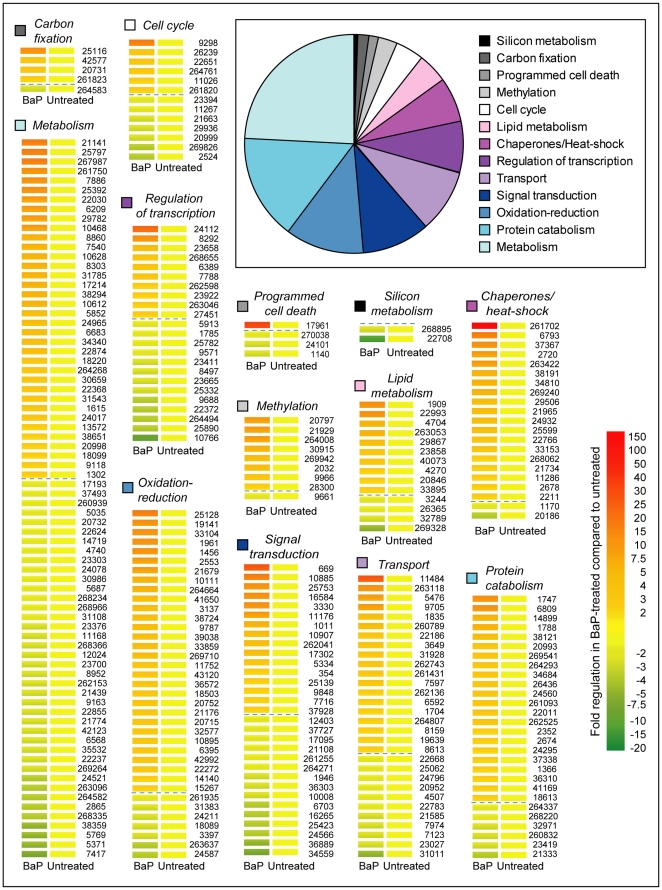
Regulation of diatom gene expression by exposure to BaP. Inset shows a pie chart with the distribution of regulated genes with available annotation [19] in functional groups. Approximately 50% of the regulated genes were annotated. Heat maps show gene expression responses induced by BaP treatment seen in the microarray, for all the genes included in the functional groups of the inset, identified by the gene specific ID number. Results are represented as average values in fold-regulation of BaP-treated samples, relative to the untreated control. Dash lines separate up- from down-regulated genes for clarity.

Functional classification was possible for about half of the regulated genes according to the available gene annotation for this species [19]. The different pathways are displayed in Figure 1. The most regulated genes were involved in metabolism (76), protein catabolism (46), oxidation-reduction (37) and signal transduction (31). Interestingly, the number of regulated genes identified in this study were 30 times higher than the number of regulated proteins identified previously in a quantitative proteomics study under the same exposure conditions to BaP [20] (653 genes compared to 21 proteins.

In addition, of the 653 genes regulated by BaP treatment, 216 genes (33%) had available Gene Ontology (GO) categories assigned [19]. From these, we found an enrichment of genes in some GO biological processes, with 8 up-regulated and 9 down-regulated categories (Table S2). The most significant up-regulated GO processes involve responses to protein damage such as proteolysis (21 genes), ubiquitin-dependent (5 genes) and independent protein catabolic processes (2 genes) and protein folding (7 genes). Several chaperones/heat-shock proteins were up-regulated with the highest regulation level for two low molecular weight chaperones HSP20 (with gene ID 261702 and 6793, with an up-regulation of 147 and 17 fold, respectively), as well as genes coding for HSP70 and HSP60. Other over-represented GO processes included electron transport (30 genes) and transport (17 genes). Additionally numerous trans-membrane transport channels showed pronounced up-regulation, in particular a potassium channel with 24 fold change respect to the control, several ABC transporters, the highest with a 8 fold regulation and a predicted Co/Zn/Cd cation transporter (3.8 fold), among others.

The most significant down-regulated GO processes included cell cycle regulation (6 genes), cell differentiation (2 genes), and DNA-dependent regulation of transcription (25 genes).

Furthermore, most of the genes involved in methylation, oxidation-reduction and carbon fixation were up-regulated (Table S1). By contrast, the two silicon metabolic genes identified in our list were down-regulated including a silicon transporter (sit1) and a silaffin precursor (Table S1).

Programmed cell death seems to be regulated in the present study with four genes showing altered expression. These include the anti-apoptotic transmembrane BAX inhibitor (tmbi) 31 fold up-regulated), as well as the down-regulation of a metacaspase (−2.1 fold), filaggrin (−2.7 fold) and a gene homologue (−2.7 fold) to a vascular associated death 1 in *Arabidopsis thaliana* [21].

Finally, we compared the present data with previous studies showing gene expression changes in the same organism due to the effects of different stress conditions including nutrient limitation, temperature and pH effect [22] as well as copper and hydrogen peroxide exposure [23]. From this analysis, we could identify several common regulated genes that could be markers of a general stress response (Table S1), including three genes, regulated under copper-toxicity conditions, were also regulated by BaP treatment (Table S1).

### Quantitative real time PCR

**Figure 2:**
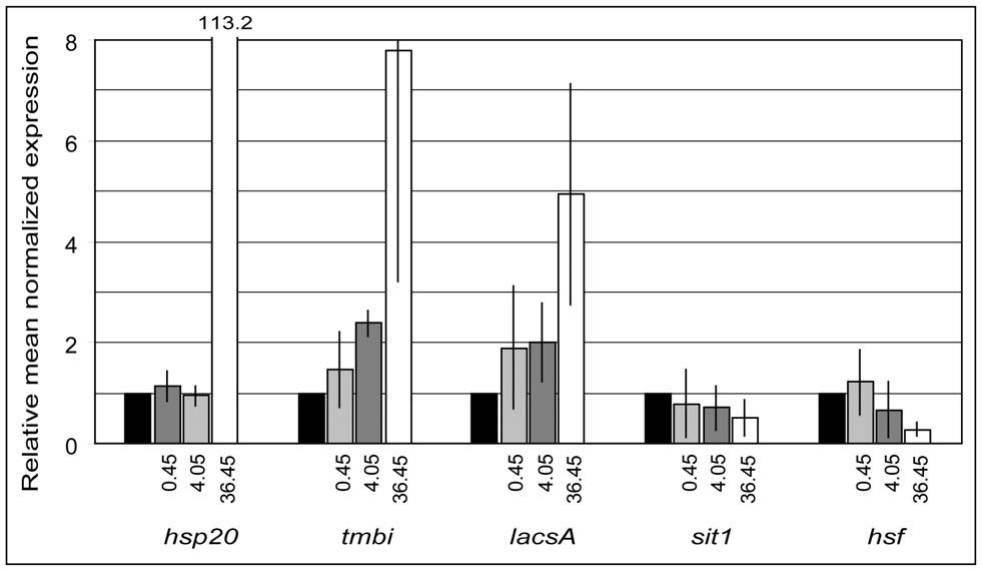
Dose response of BaP and gene expression analysis of selected genes by qRT-PCR. The expression of genes relative to the house-keeping gene *gapdh* was analyzed from diatom cultures exposed for 48 h to BaP at the concentrations 0.45 µg/L (dark grey), 4.05 (light grey) and 36.45 µg/L (white), and normalized against the solvent control samples (black bars). Numbers above bars correspond to the out of scale fold-change values. Bars represent the average fold-regulation of the genes with vertical lines representing the standard deviation of the mean.

To confirm the microarray results, we have selected six genes from different biochemical pathways that showed pronounced regulation by DNA microarray and performed qRT-PCR. Thus, three genes showing up-regulation were tested, a small sized-heat shock protein (hsp20), a putative transmembrane BAX inhibitor (tmbi) and a long-chain acyl-CoA synthase (lacsA) (ID numbers 261702, 17961 and 29867, respectively). In addition we also tested three genes showing down-regulation including silicon transporter 1, 4-hydroxyphenylpyruvate dioxygenase (diox) and a heat shock transcription factor (hsf) (ID numbers 268895, 5371 and 10766, respectively). Using the same BaP concentration and time of exposure as in the microarray, we were able to confirm the regulation of all the selected genes by qRT-PCR (Figure S1). The regulation was observed not only using the total RNA extracted from the biological samples used in the microarray but also in additional biological replicates that had been treated in the same way [24]. Upon treatment of diatom cultures with decreasing amounts of BaP (36.45, 4.05 and 0.45 µg/L) and higher exposure times (48 h), up-regulation of the lacsA and the tmbi gene expression was still evident, as well as down-regulation of sit1 and the hsf. However, no regulation of the hsp20 could be observed at BaP concentrations lower than 36.45 µg/L (Figure 2). Finally, regulation of some of these genes was also found when diatom cultures were exposed to surface sediments contaminated with PAHs [24].

## Discussion

The present data is part of a wider effort in our group in the identification of biomarkers to serve as bioindicators of general stress conditions as well as markers of exposure to specific substances. Some of the substances are the polycyclic aromatic hydrocarbons with a wider distribution in aquatic environments and that are of major concern upon acute toxic peaks of pollution caused by oil spills.

**Figure 3:**
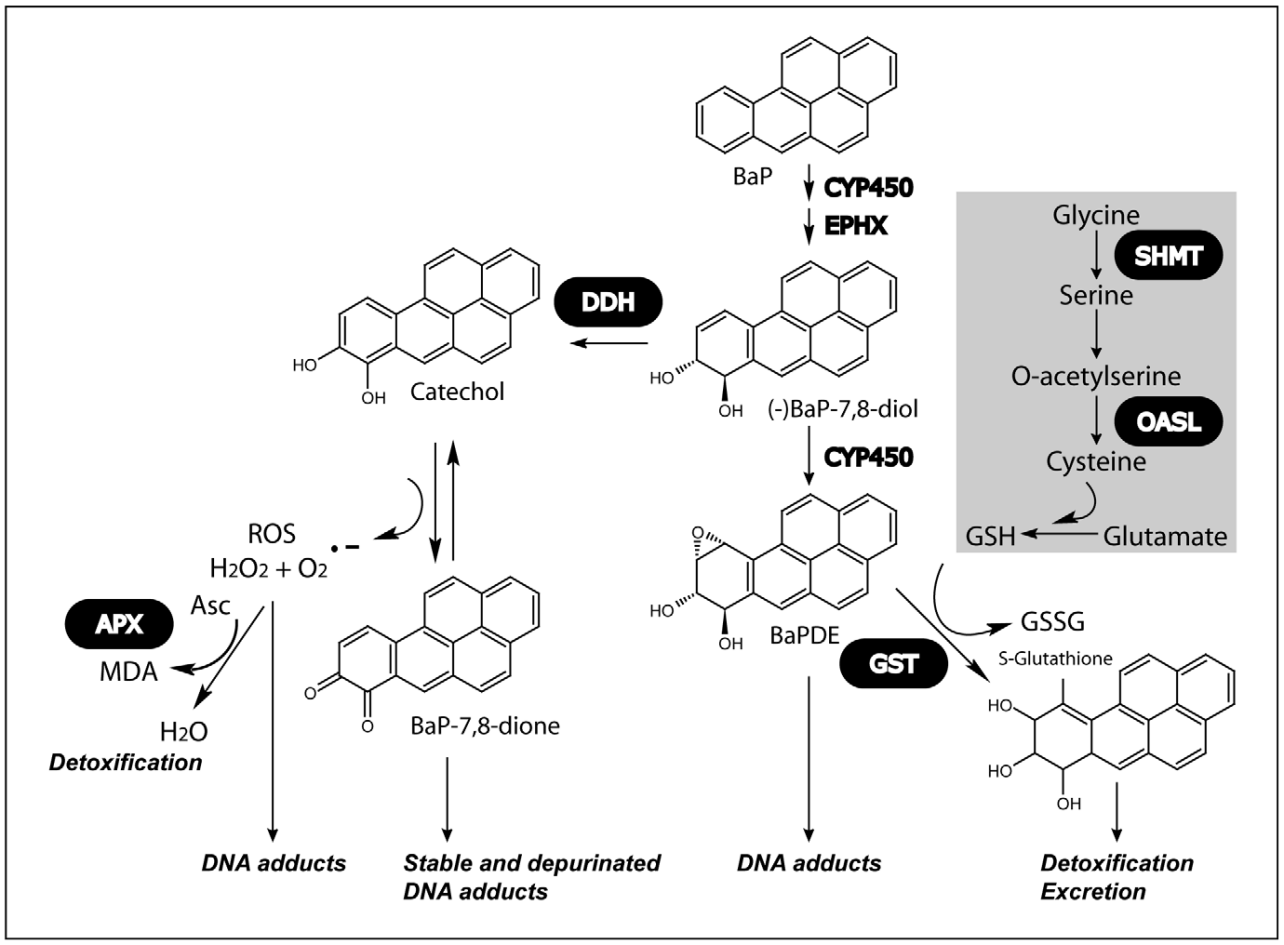
Pathways for BaP metabolism and detoxification. BaP is metabolized into BaP-7,8-diol by cytochrome P450 (CYP450) and epoxide hydroxylase (EPHX) in a two-step reaction. BaP reactive metabolites, such as BaP-7,8-diol-9,10 epoxide (BaPDE) can form adducts with DNA, causing mutagenesis. The reactive metabolites generated can be inactivated via conjugation with glutathione (GSH) by glutathione S-transferase (GST). Reactions involved in glutathione synthesis for which up-regulated proteins were identified by BaP treatment are shown inside the grey box and include Serine hydroxymethyltransferase (SHMT) and O-acetylserine lyase (OASL). Alternatively, BaP-7,8-diol can be converted to catechol by a dihydrodiol dehydrogenase (DDH), which can be oxidized to BaP-7,8-dione. Produced reactive oxigen species (ROS) can be detoxified by the action of several enzymes, including ascorbate peroxidase (APX). Enzymes coding genes up-regulated in BaP-exposed diatoms are shown in a black box.

PAHs are ubiquitous environmental pollutants showing carcinogenic potency after undergoing metabolic activation. Cytochrome P450 and epoxide hydrolase are known to catalyse the conversion of BaP into epoxides [25], which can bind chemically to DNA and exert potent mutagenic and carcinogenic effects (Figure 3). However, our microarray data could not identify regulated genes coding for cytochrome P450 or epoxide hydroxylase, as assigned from homology alignments with other species [19], which lead us to suggest that other proteins could be involved in BaP metabolism in diatoms. Nevertheless, we were able to identify other BaP-metabolizing genes regulated in our microarray study. It has been shown that the dihydrodiol dehydrogenase enzyme (an aldo-keto reductase) can afford protection against the formation of epoxides resulting from BaP-metabolism, by sequestering the *trans*-dihydrodiol compound and catalyzing its oxidation to redox active ortho-quinones (Figure 3) [26], [27]. This alternative pathway for the activation of BaP seems to take place under our experimental conditions, where a 3 fold up-regulation of dihydrodiol dehydrogenase gene (ID 36572) (Table S1) is observed. A similar up-regulation of human dihydrodiol dehydrogenase has been observed in HepG2 hepatoma cells in response to BaP or reactive oxygen species (ROS) [28]. The formation of ROS seems to be a general outcome of BaP metabolism and the induced oxidative stress can affect cells in different ways including protein oxidative damage, DNA damage and lipid peroxidation.

Ascorbate peroxidase (APX, with gene ID 38724) is a detoxifying enzyme that is up-regulated in BaP-exposed diatom and converts hydrogen peroxide to water. In addition, the conjugation of BaP reactive metabolites, in particular BaP-7,8-diol, with glutathione (GSH) by glutathione S-transferase (GST), can inactivate this compound and prevent the formation of adducts with DNA and mutagenesis [29]. Therefore, an increased expression of GST is generally observed upon cellular stress and is also observed in this study (gene ID 5852), while additional genes involved in the metabolism of glutathione also show an increase in expression (Figure 3).

### Stress response

**Figure 4:**
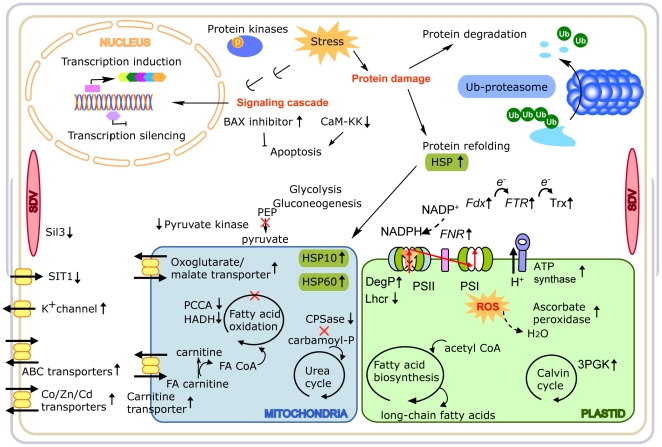
Cellular pathways involved in the diatom response to BaP treatment at sub-lethal concentrations. Regulated pathways include transcription regulation, protein refolding, protein degradation, trans-membrane transport, lipid metabolism and silicon metabolism, among others. Arrows next to specific genes indicate an up- or down-regulation observed on the microarray.

Our data shows an extensive up-regulation of genes linked with an oxidative stress response, and a general cellular response to overcome stress-induced damage, particularly protein damage. Thus, in addition to genes involved in oxidation-reduction there is a significant amount of up-regulated genes linked with protein refolding, like heat-shock proteins/molecular chaperones or involved in protein degradation, including the ubiquitin/proteasome system (Table S1; Figure 4). This is consistent with a BaP-induced stress scenario, in which there is some level of cellular damage triggering repair mechanisms while apparently not being acute enough to induce cell death. Thus, there is significant increase in the expression of a putative transmembrane BAX inhibitor gene, an inhibitor of Bcl-2-induced cell death [30]) and a decrease in the expression of genes coding for a filaggrin and a vascular associated cell death 1 proteins, which have been linked to programmed cell death. A putative metacaspase, with a conserved C14 caspase domain was also down-regulated (with ID 270038). Metacaspases are widespread among prokaryotic and eukaryotic phytoplankton and the expression of some metacaspases have been linked to programmed cell death events in *Thalassiosira pseudonana* such as reduced photosynthetic efficiency, caspase activation and cell mortality [31]. However, the metacaspase identified as down-regulated in this study has been described previously as being constitutively expressed at the gene and protein levels [31] and its role if any in programmed cell death events is unknown.

The observation of thirteen genes involved in cell cycle regulation, including the decreased expression of many cyclins (Table S1), suggests an arrest in the cell cycle progression probably to allow the repair of any BaP-induced damage. This is consistent with the decreased diatom growth rate observed under exposure to BaP in the present study (data not shown) as well as in previous studies [20], [32].

Significant protein misfolding seems to occur as a result of BaP exposure, as inferred from parallel cellular responses that either fate the damaged molecules to be refolded by molecular chaperones or to enter into proteolytic pathway when the damage is beyond repair. We observed an increase in expression of 18 genes belonging to the chaperone heat-shock response out of 20 regulated genes. Several genes were highly expressed, including a low molecular weight heat shock protein hsp20 (Figure S1; Table S1), while several other HSPs show lower but significant up-regulation (Table S1). However, upon treatment with lower concentrations of BaP, which cause no significant growth inhibition in diatoms, no regulation was observed for hsp20, which suggests that the increased expression of this protein is only required at concentrations causing significant stress in diatoms.

In addition, an increased expression of peptidyl-prolyl cis-trans isomerase B precursor genes (with ID 37367 and 33153) was observed in our study, which is consistent with the up-regulation observed for this protein in MCF-7 cells treated with BaP, where also a HSP70 was among the proteins showing the highest fold increase [33].

Another protein previously described as being involved in the response to various stress conditions such as oxidation, salt, pH and heat is the DegP protease found in most organisms. Bacterial DegP has been shown to combine molecular chaperone and proteolytic functions, with the activity being controlled by a temperature-dependent switch [34]. In addition, a chloroplast DegP protease in *Arabidopsis thaliana* was described to be involved in the damage and repair events of photosystem II in photoinhibited plants by performing the initial degradation of the photodamaged D1 protein [35]. Two genes putatively coding for DegP proteases (with ID 14899 and 264293) showed an increased expression upon BaP treatment by 6 and 3 fold, respectively. As occurs in other organisms, an increase in DegP expression seems to be a general response in diatoms towards environmental stress by contaminants, since an up-regulation of this gene has been observed as well by DNA microarray upon diatom exposure to the fungicide flusilazole (Carvalho & Lettieri, unpublished data).

Interestingly, we found that three genes previously described as being induced by copper stress in *T. pseudonana* [23], are also regulated by BaP-exposure. Two of such genes in particular, called *cue 7* and *cue 6* (with the ID numbers 264384 and 269876, respectively), were reported as being strongly induced by increasing concentrations of copper, in particular for short-term exposures, but not significantly by cadmium exposure or H_2_O_2_ [23]. For that reason they were suggested to correlate their expression to a Cu-associated response. However, these genes showed a strong up-regulation in our BaP-exposed diatom cultures, and a down-regulation upon conditions of iron limitation [36]. This indicates a more general involvement of these genes in the cellular response to stress conditions than previously believed [23]. By contrast, we found a slight down-regulation for the gene with ID number 269696, which has a 90% identity with a previously reported copper stress-related *cue 4/5* gene. The expression of the *cue 4/5* gene was found to be induced by copper by 2 fold and by H_2_O_2_ by 5 fold for long-term exposures of 24 h, while for short-term exposures (1 h) it showed no effect for copper and a reduced expression for H_2_O_2_ exposure [23]. In addition it showed a down-regulation upon conditions of nutrient limitation, including silica, iron and nitrogen [22]. Thus, it seems that the regulation of this gene with unknown function could be related with a conserved short-term stress response.

### Lipid metabolism

Due to their hydrophobic nature, PAHs in the medium readily incorporate into cellular membranes [37], where they can immediately exert some of their cytotoxic actions, in addition to the most recognized long-term carcinogenicity effects caused by PAH metabolization products. Studies on the interaction between BaP and phosphatidylcholine membranes have shown a rapid miscibility and homogeneous distribution of BaP in the hydrophobic region, establishing strong hydrophobic interactions with the phospholipid acyl chains, as also confirmed from a simulated interaction between BaP and a DMPC bilayer [38]. This localization results in an expansion and swelling of the membrane and is expected to perturb the membrane integrity and functionality [38]. In addition, BaP was also shown to modulate cholesterol and fatty acid content in organized membrane domains [39].

We have found fourteen regulated genes involved in the lipid metabolic pathway, ten showing up-regulation and four showing down-regulation of expression (Figure 1; Table S1). Three of the down-regulated genes are mostly involved in lipid catabolism. The up-regulated genes include genes involved in the phospholipid synthesis, including glycerophospholipids and long-chain phospholipids. Overall, our data suggest that BaP exposure to diatom induce a response consisting both of increased lipid synthesis and decreased lipid degradation, which could serve to restore the membrane organization affected by BaP incorporation (Figure 4). Of particular interest, the gene with ID number 29867, coding for a long chain acyl-coA synthetase (lacsA) is up-regulated by 4 fold by BaP in the present DNA microarray study (Table S1) and 14 fold by qRT-PCR (Figure S1) and has been shown previously by our group to be up-regulated both at the gene and protein expression levels upon diatom exposure either to BaP or to a PAH mixture, including contaminated sediment extracts [24], [32]. This enzyme is involved in the activation of long-chain fatty acids, and is particularly active towards C20 and C22 polyunsaturated fatty acids [40]. The increased expression of this gene and the translated protein seem to be correlated mainly with BaP concentration and not with the total concentration of PAHs present upon exposure to diatom, which has led us previously to suggest lacsA as a biomarker for BaP exposure [24].

### Silicon metabolism

Silicon metabolism in the diatom *T. pseudonana* has been one of the first identified pathways affected by PAHs in general and BaP in particular, with genes involved in the silica shell formation showing regulation upon treatment conditions by qRT-PCR [32]. In the present study, two genes belonging to this pathway showed a decrease in expression, coding for silicon transporter 1 (sit1) and a silaffin precursor with a high homology to silaffin 3 (ID numbers 268895 and 22708, respectively) (Table S1). Silaffin 3 has been shown previously by qRT-PCR to be down-regulated upon BaP treatment [32] as well as upon exposure to contaminated surface sediment extracts [24]. Sit1 has been recently identified by a quantitative proteomics approach as being down-regulated upon BaP treatment [20] and a similar regulation was induced in the presence of PAH-contaminated marine surface sediments [24]. In addition, by qRT-PCR, the fold-regulation of sit1 was observed even at lower concentrations of BaP treatment (Figure 2).

Analysis of the different silicon transporter levels across the cell cycle has shown that their protein expression correlate with periods of silica incorporation into cell wall structures [41]. However, mRNA levels did not correlate with protein levels, and indeed they observed a peak in mRNA during the S-phase when no silicification occurs, and the SIT protein levels were minimal [41]. By contrast, the decreased level of sit1 induced by BaP treatment is observed both at the mRNA and protein levels and has been linked to impairment in the silicon uptake by the diatom cells [20]. Despite SIT1 and SIT2 sharing 88% amino acid identity, and their expression following the cell cycle in a similar way [41], no regulation was observed for SIT2 in our microarray and protein analysis [20], suggesting that their expression is regulated and affected differently by BaP treatment.

### Conclusions

We used a transcriptomics approach to study the effect of BaP on a marine diatom and identified the mode of action of this persistent pollutant. At sub-lethal concentrations, the highly hydrophobic BaP triggers a change in the lipid metabolism of the cell, probably in response to its incorporation and perturbation of cellular membranes. In parallel, apoptosis is inhibited and the normal progression of the cell cycle is disrupted by either regulating cyclin-like proteins or by decreasing the available silica in the cells. Stress-induced damage on proteins seems to be one of the main targets of the cellular efforts together with the detoxification of carcinogenic BaP metabolites and ROS.

Overall, the methodology was able to identify molecular markers for the general stress response in this organism as well as regulated pathways linked with the chemical properties of the pollutant. Furthermore, we observed a dose-dependent response of selected genes, which supports the use of the identified biomarkers in field samples, where lower concentrations of PAHs are usually present. Because diatoms share many molecular features to both animal and plants, the molecular biomarkers identified in this study are likely conserved in higher organisms allowing a comparative analysis along the trophic levels.

## Materials and Methods

### Diatom strain and routine culture

*Thalassiosira pseudonana* (strain CCMP 1335) was obtained as axenic culture from the Provasoli-Guillard National Center for Culture of Marine Phytoplankton (CCMP, West Boothbay Harbour, Maine, USA). Diatoms were maintained at 6–8°C under a diurnal light cycle of 13 h light and 11 h darkness. The culture medium was f/2-medium (Guillard, 1975) based on 3.2% artificial sea water (ASW, Sigma-Aldrich, Steinheim, Germany). *T. pseudonana* was cultured in 250 mL Erlenmeyer flasks at densities between 0.5×10^6^–2.5×10^6^ cells/mL. Doubling times under these conditions were ca. 24 h. Fresh cultures for maintenance were inoculated every seven days.

### Exposure of *T. pseudonana* to Benzo[a]pyrene (BaP)

*T. pseudonana* was exposed to the Polycyclic Aromatic Hydrocarbon (PAH) BaP to detect effects of this compound on growth and on gene expression. Cell densities were determined over 24 h of exposure with BaP using photometric measuring as described previously [32]. Growth rates were calculated from cell densities and used to determine growth inhibition compared to the control. Diatom cultures from control (exposed to the methanol solvent) and BaP-treated conditions inducing around 40% growth inhibition (36.45 µg/L) were harvested for gene expression analysis, in four biological replicates. For dose-response analysis, *T. pseudonana* was exposed for 48 h to BaP concentrations of 0.45, 4.05, and 36.45 µg/L.

### Probe and microarray design

60mer probes for spotting on the array were designed using the software Array Designer 4.0 (Premier Biosoft International, USA) for the whole transcriptome available from JGI Thaps 3.0 database. For finding probes with appropriate design for the Agilent standard protocols, the Array Designer default values were applied, with the exception of the probe length with default 60mer probes (60±0 nucleotides) and a melting temperature of 80±5°C. Where no probe could be identified under the default conditions, the range of the default values was widened; this accounted for <1% of the probes. Using these conditions, a total of 11 402 different probes were designed corresponding to the initial *T. pseudonana* transcriptome available on JGI database 3.0 [14], 984 were rated with best quality, 9889 with good quality and 529 with poor quality.

In addition, hybridization controls for artificial RNA mixed with the samples for quality control (Spike In Kit, Agilent), were added on the array following the standard Agilent protocol. The array design was submitted to the company (Agilent, USA) which manufactured the 8×15K microarray using their standard on slide in situ synthesis technique. Probes were randomly distributed across the array. The array design was submitted to ArrayExpress (A-MEXP-2128).

### Total RNA isolation and preparation of cRNA for microarrays

RNA extraction was performed using the Trizol LS (Invitrogen) method according to the manufacturer's protocol with small modifications to improve the yield [32].

1 µg of total RNA was used for transcription. Sample preparation was performed using the Agilent One color low RNA input linear Amplification Kit and the standard protocol. Thus, to the 1 µg of RNA sample, each 5 µL of diluted hybridization control (Agilent One Color RNA Spike-In Kit according to Agilent standard protocol) were added. Then total RNA was transcribed to cDNA, which was then in one step transcribed to cRNA and labeled with Cy3. Yields of cRNA prepared from 1 µg of total RNA varied between 5 and 13 µg. Quality of total RNA and labeled cRNA was checked using the Bioanalyzer. Furthermore, labeling quality was checked on NanoDrop to assess the ratio of pmol dye/ng cRNA. Cy3-ratios ranged from 9.0 to 15.5, so that all samples fulfilled the manufacturer's recommendation to use only samples with a ratio >8.

### Hybridization and scanning of microarray

600 ng of Cy3-labeled cRNA samples were hybridized on the customized microarrays according to the Agilent standard protocol. Raw data processing was performed using the Agilent Scan Control and the Agilent Feature Extraction Software, according to the manufacturer's instructions. The hybridization controls showed always good quality, i.e. linearity for the different concentrations used in the spike-in kit.

### Microarray data evaluation

Data from feature extraction were imported into Agilent Gene Spring 10 for data evaluation and normalized using the Gene Spring algorithm for 1 color microarrays. Data filtering was performed, including flag filtering for those signals which were present or marginal in at least 4 out of 8 samples, and filtering on expression level to exclude all signals which were regulated less than 2 fold compared to the treatment. Among the remaining genes, those significant in a T-Test at a level of p<0.05 and which passed the Benjamin-Hochberg Multiple Testing Correction were selected. Data is compliant to MIAME guidelines.

Six genes with high and statistically significantly regulation on the microarray, and representing different pathways, were chosen for further investigation using quantitative Real-Time Polymerase Chain Reaction (qRT-PCR).

### Quantitative Real-Time PCR

Total RNA from diatom cultures exposed to 36.45 µg/L for 24 h or to different BaP concentrations for 48 h were used for transcriptomics analysis by qRT-PCR with selected target genes. Each 2 µg of RNA were reversely transcribed to cDNA using oligodT primers and SuperScript II Reverse Transcriptase Kit as recommended (Invitrogen). Primers and MGB TaqMan probes for qRT-PCR, were designed using Primer Express® Software and synthesized by Applied Biosystems (Foster City, CA, USA). All sequences for primers can be found in Table 1. qRT-PCR was performed using the ABI Prism 7900HT sequence detection system (Applied Biosystem) as detailed [32]. Gene expression data from qRT-PCR were evaluated using Q-Gene [42], which takes into account the amplification efficiencies of target and reference genes. For all qRT-PCR results, *gapdh* was used as housekeeping gene. *gapdh* threshold cycles for amplification in qRT-PCR did not change significantly from each other for none of the different treatments (One way ANOVA). The calibrator sample was the control sample containing only the carrier solvent methanol (termed 0).

**Table 1:** Genes analyzed by qRT-PCR.

Name of gene	Protein ID	Forward primer	Reverse primer	TaqMan Probe 6-FAM-sequence-MGB	Cellular processes
Low molecular weight heat shock protein	261702	CGAAAAGTGAGGATGAAGGAATCT	TTCCTGTCGAGGATGAATGAATT	CGTACACTCTCGTTTCGA	Chaperone/heat-shock response
Transmembrane BAX inhibitor motif-containing	17961	CTCCTTGCGACGTTTACGATT	GCCGTGGTGGTGGTACCA	TCGAGGCGTTTCTT	Programmed cell death
long-chain acyl-CoA synthetase	29867	GGCATGTCGTGTGTGGTTTG	TTGGCCTCGCACAATCG	TCGAGGAAGGAGTTGGA	Lipid metabolism
Silicon transporter 1	268895	TTGCCGAGGATGCCTAAACTT	TGACGAGCTACTGCAGGTTCA	TGGCAATCTGTTGTAAAT	Silicon metabolism
4-hydroxyphenylpyruvate dioxygenase	5371	ATGGACGCCCGTTGGAT	TGAAAATGCAATGATTCCCAAA	TCAAACGAGGCCAAAAG	Metabolism
heat shock transcription factor	10766	GGTTGGTGCATCTCAAATTGG	TTGCATGTAGCTGTGGCAAAG	AGCACACCAGCGATG	Regulation of transcription

All primers are listed from 5′to 3′.

## Supporting Information

Figure S1 **Gene expression analysis of selected genes by qRT-PCR.** The expression of genes relative to the house-keeping gene gapdh was analyzed from diatom cultures exposed to 36.45 ug/L BaP (grey bars), and normalized against the solvent control samples (white bars) with a 24 h exposure. For comparison, the fold-change gene expression from the microarray experiments is represented as black bars. Numbers above bars correspond to the fold-change values with respect to the control. Bars represent the average calculated from at least three individual experiments with vertical lines representing the standard deviation of the mean. Asterisks label those genes for which there was a significant difference between the BaP-exposed sample and the solvent control by qRT-PCR (t-test, p<0.05).
(TIF)

Table S1 **Genes regulated in exponentially growing** ***T. pseudonana*** **cells exposed to benzo[a]pyrene.**
(DOC)

Table S2 **GO biological processes that are regulated by BaP in the diatom** ***T. pseudonana*** **.**
(DOC)
