# Supplementary figures and images for: Transcriptomics Responses in Marine Diatom Thalassiosira pseudonana Exposed to the Polycyclic Aromatic Hydrocarbon Benzo[a]pyrene
Source: PLoS One. 2011 Nov 3;6(11):e26985. doi: 10.1371/journal.pone.0026985 (PMC3207822; doi:10.1371/journal.pone.0026985)

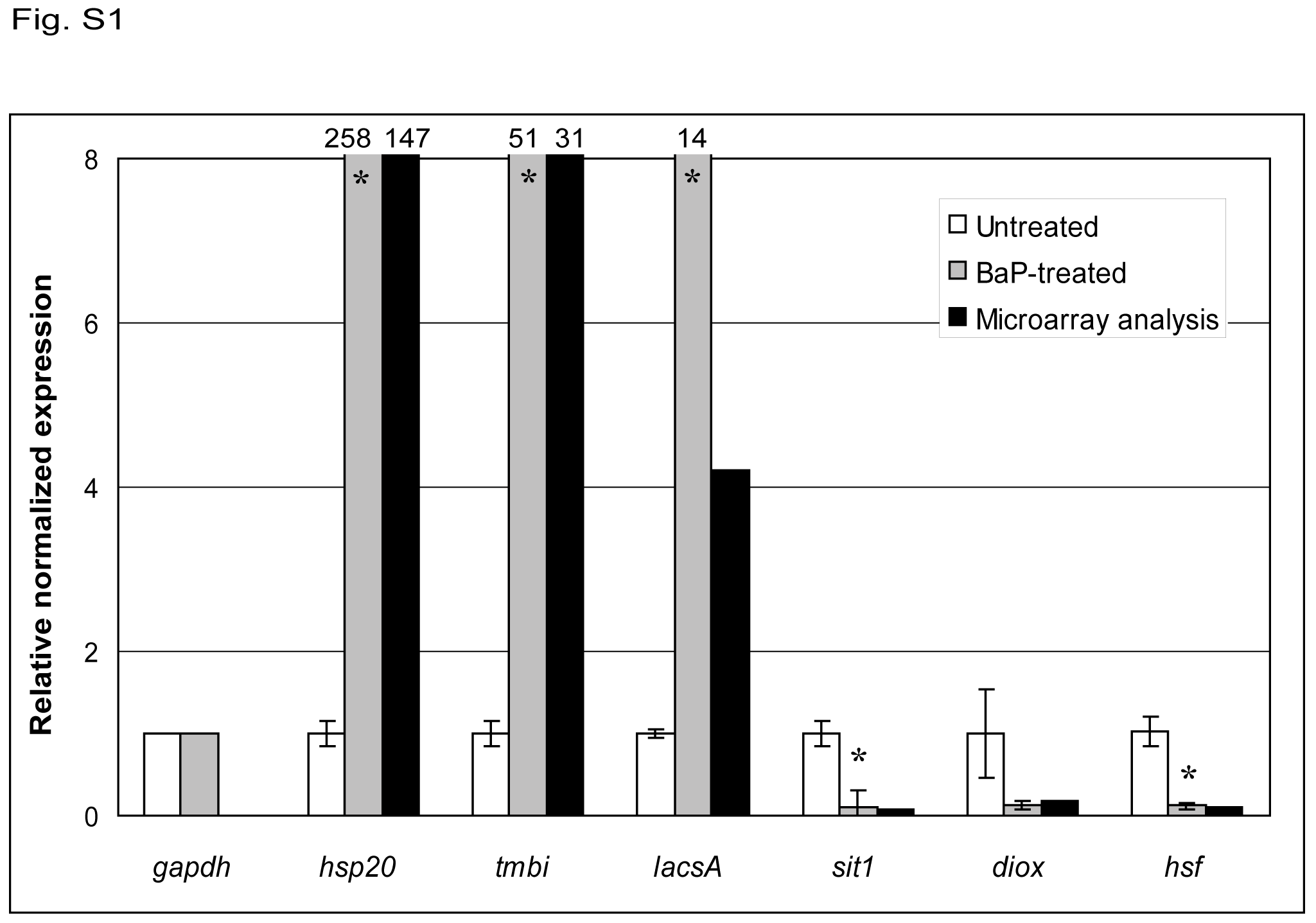

Supplement: Figure S1 — Gene expression analysis of selected genes by qRT-PCR. The expression of genes relative to the house-keeping gene gapdh was analyzed from diatom cultures exposed to 36.45 ug/L BaP (grey bars), and normalized against the solvent control samples (white bars) with a 24 h exposure. For comparison, the fold-change gene expression from the microarray experiments is represented as black bars. Numbers above bars correspond to the fold-change values with respect to the control. Bars represent the average calculated from at least three individual experiments with vertical lines representing the standard deviation of the mean. Asterisks label those genes for which there was a significant difference between the BaP-exposed sample and the solvent control by qRT-PCR (t-test, p<0.05). (TIF) [file pone.0026985.s001.tif]
