# Supplementary material for: Transcriptomics Responses in Marine Diatom Thalassiosira pseudonana Exposed to the Polycyclic Aromatic Hydrocarbon Benzo[a]pyrene
Source: PLoS One. 2011 Nov 3;6(11):e26985. doi: 10.1371/journal.pone.0026985 (PMC3207822; doi:10.1371/journal.pone.0026985)
Supplement: Table S1 — Genes regulated in exponentially growing T. pseudonana cells exposed to benzo[a]pyrene. (DOC) [file pone.0026985.s002.doc]

**Table S1.** Genes regulated in exponentially growing *T. pseudonana* cells exposed to benzo[a]pyrene.

| **Protein ID** | **Species** | **Function^a^** | **Fold change** | **Fold change in other studies^b,c,d^** |
| --- | --- | --- | --- | --- |
| **Oxidation-reduction** | | | | |
| 25128 | *Strongylocentrotus purpuratus* | PREDICTED: similar to nucleoredoxin | +14.58 |  |
| 19141 | *2nd Hit: ACC No. YP_291988 Prochlorococcus marinus str. NATL2A* | 2nd Hit flavodoxin, long chain | +11.74 | (+3.36 T)^b^ |
| 33104 | *Nostoc sp. PCC 7120* | D-lactate dehydrogenase | +10.86 |  |
| 1961 | *Azoarcus sp. EbN1* | s5h: putative salicylate monooxygenase | +10.74 |  |
| 1456 | *Chlorobium phaeobacteroides BS1* | Cphamn1DRAFT_2922: Isocitrate dehydrogenase NADP-dependent, monomeric type | +9.45 |  |
| 2553 | - | Hypothetical Proteins No BLAST results-PFAM: Structure-specific recognition protein 21253.4kDa | +7.52 |  |
| 21679 | *Synechocystis sp. PCC 6803* | (bacterial type ferredoxin family) | +6.57 |  |
| 10111 | *Thermotoga maritima MSB8* | oxidoreductase, putative | +4.62 |  |
| 264664 | *Hahella chejuensis KCTC 2396* | thioredoxin | +4.58 |  |
| 41650 | *Roseovarius nubinhibens ISM* | choline dehydrogenase | +4.46 |  |
| 3137 | - | Hypothetical Proteins No BLAST results-PFAM: Oxidoreductase family, NAD-binding Rossmann fold | +4.27 |  |
| 38724 | *Euglena gracilis* | ascorbate peroxidase | +4.25 | (-3.08 Si)^b^ |
| 9787 | *Oryza sativa (japonica cultivar-group)* | putative prolyl 4-hydroxylase | +4.00 |  |
| 39038 | *Plasmodium berghei strain ANKA* | hypothetical protein | +3.97 |  |
| 33859 | *Strongylocentrotus purpuratus* | PREDICTED: similar to nicotinamide nucleotide transhydrogenase | +3.90 |  |
| 269710 | *Dictyostelium discoideum* | hypothetical protein DDB0168923 | +3.51 |  |
| 11752 | *3nd Hit: ACC No. YP_237919 Pseudomonas syringae pv. syringae B728a* | 3nd Hit:2OG-Fe(II) oxygenase superfamily | +3.27 |  |
| 43120 | *Medicago truncatula* | Thioredoxin, putative | +3.12 |  |
| 36572 | *Phytophthora infestans* | dimeric dihydrodiol dehydrogenase, putative | +3.09 |  |
| 18503 | *Plasmodium berghei strain ANKA* | thioredoxin-related protein | +2.91 |  |
| 20752 | - | Hypothetical Protein No BLAST result; PFAM: Thioredoxin | +2.88 |  |
| 21176 | *Gibberella zeae PH-1* | hypothetical protein FG09695.1 | +2.81 |  |
| 20715 | *Zea mays* | thiol oxidoreductase | +2.78 |  |
| 32577 | *Methanococcoides burtonii DSM 6242* | Aspartate-semialdehyde dehydrogenase, USG-1 related | +2.74 |  |
| 10895 | *Arabidopsis thaliana* | disulfide oxidoreductase/ electron carrier | +2.67 |  |
| 6395 | *Flavobacterium johnsoniae UW101* | Monooxygenase, FAD-binding | +2.65 |  |
| 42992 | *Strongylocentrotus purpuratus* | PREDICTED: hypothetical protein XP_780404 | +2.65 |  |
| 22272 | *Plasmodium falciparum 3D7* | hypothetical protein PFB0115w | +2.53 |  |
| 14140 | *Arabidopsis thaliana* | ATPDIL5-4; electron transporter | +2.42 |  |
| 15267 | *Nostoc punctiforme PCC 73102* | COG1453: Predicted oxidoreductases of the aldo/keto reductase family | +2.23 |  |
| 24587 | *Acidobacteria bacterium Ellin345* | Homoserine dehydrogenase | -4.14 |  |
| 263637 | *Arabidopsis thaliana* | oxidoreductase | -3.95 |  |
| 3397 | *Dictyostelium discoideum AX4* | DDBDRAFT_0206234: hypothetical protein | -3.02 |  |
| 18089 | *Toxoplasma gondii* | peroxidoxin 2 | -2.81 |  |
| 24211 | *Flavobacteriales bacterium HTCC2170* | NADH:flavin oxidoreductase, Old Yellow Enzyme family protein | -2.72 |  |
| 31383 | *Odontella sinensis* | glyceraldehyde-3-phosphate dehydrogenase precursor | -2.53 | (-3.61 Si; -3.43 N)^b^ |
| 261935 | *Burkholderia vietnamiensis G4* | Thioredoxin | -2.45 |  |
| **Programmed cell death** | | | | |
| 17961 | *Xenopus tropicalis* | MGC88883 protein  putative transmembrane BAX inhibitor motif-containing protein | +31.33 |  |
| 24101 | *Homo sapiens* | filaggrin | -2.66 |  |
| 270038 | *3nd Hit: ACC No. AAL87229 Acanthamoeba castellanii* | 3nd Hit:metacaspase | -2.08 |  |
| 1140 | *Arabidopsis thaliana* | VAD1 (vascular associated death1) | -2.71 |  |
| **Cell cycle** | | | | |
| 9298 |  | Hypothetical Protein No BLAST result; PFAM: Cyclin | +13.81 |  |
| 26239 | *Arabidopsis thaliana* | unknown protein | +3.19 |  |
| 22651 | *2nd Hit: ACC No. NP_173083 Arabidopsis thaliana* | 2nd Hit CYCB3;1; cyclin-dependent protein kinase | +2.75 |  |
| 264761 | *Gallus gallus* | DNA ligase IV | +2.54 |  |
| 11026 | - | Hypothetical Protein No BLAST result; PFAM: Cyclin | +2.34 |  |
| 261820 | *Strongylocentrotus purpuratus* | PREDICTED: similar to cyclin-dependent kinase 10 isoform 1 | +2.27 | (+3.27 Si)^b^ |
| 2524 | *Medicago truncatula* | MtrDRAFT_AC146856g11v1: Cyclin, N-terminal | -3.83 |  |
| 269826 | *Apis mellifera* | PREDICTED: similar to ENSANGP00000021921 | -3.79 |  |
| 20999 | *Hydra viridis* | cyclin A | -2.98 |  |
| 29936 | *Nicotiana tabacum* | MCM protein-like protein | -2.79 |  |
| 21663 | *-* | - | -2.77 |  |
| 11267 | *Mus musculus* | unnamed protein product | -2.47 |  |
| 23394 | *Trypanosoma cruzi strain CL Brener* | cyclin 1 | -2.07 |  |
| **Signal transduction** | | | | |
| 669 | *2nd Hit: ACC No. XP_131323 Mus musculus* | 2nd Hit PREDICTED: similar to GTP-binding nuclear protein Ran (GTPase Ran) (Ras-like protein TC4) | +24.42 |  |
| 10885 | *Clostridium beijerincki NCIMB 8052* | Coagulation factor 5/8 type, C-terminal | +15.49 |  |
| 25753 | *Arabidopsis thaliana* | intramolecular transferase, phosphotransferases | +11.55 |  |
| 16584 | *Lotus japonicus* | Ser/Thr protein kinase | +10.80 |  |
| 3330 | *Bdellovibrio bacteriovorus HD100* | Bd1502: putative cAMP-dependent protein kinase | +10.42 |  |
| 11176 | *Rhodopirellula baltica SH 1* | conserved hypothetical protein | +6.74 |  |
| 1011 | *Arabidopsis thaliana* | AT3G23610: dual specificity protein phosphatase (DsPTP1) | +5.59 |  |
| 10907 | *Rana pipiens* | guanylate cyclase activating protein 1 | +4.41 |  |
| 262041 | *Danio rerio* | novel protein | +4.02 |  |
| 17302 | *Oceanicola batsensis HTCC2597* | Inositol monophosphatase family protein | +3.53 |  |
| 5334 | - | Hypothetical Protein No BLAST result; PFAM: Cytochrome b561 | +2.65 |  |
| 354 | *Apis mellifera* | PREDICTED: similar to ENSANGP00000020417 | +2.40 |  |
| 25139 | *Ustilago maydis 521* | hypothetical protein UM01371.1 | +2.25 |  |
| 9848 | *Tribolium castaneum* | PREDICTED: similar to CG7650-PA | +2.20 |  |
| 7716 | *Takifugu rubripes* | makorin RING finger protein 1b | +2.11 |  |
| 37928 | *Homo sapiens* | histone acetyltransferase | +2.02 |  |
| 34559 | *Cryptosporidium hominis TU502* | hypothetical protein Chro.30121 | -7.23 |  |
| 36889 | *Theileria parva strain Muguga* | calmodulin-domain protein kinase | -5.35 |  |
| 24566 | *Bos taurus* | PREDICTED: similar to TNF receptor-associated factor 3 interacting protein 1 | -4.18 |  |
| 25423 | - | Hypothetical Protein No BLAST result: ProSite- EGF-like domain signature 2 | -4.06 |  |
| 16265 | *Medicago truncatula* | EPS15 homology (EH); Protein kinase | -4.05 |  |
| 6703 | *Oceanospirillum sp. MED92* | sensory box/GGDEF/EAL/CBS domain protein | -3.82 |  |
| 10008 | *Nostoc punctiforme PCC 73102* | COG3899: Predicted ATPase | -3.17 |  |
| 36303 | *Dictyostelium discoideum* | hypothetical protein DDB0185742 | -2.58 |  |
| 1946 | *Dictyostelium discoideum* | similar to Dictyostelium discoideum (Slime mold). Homeobox-containing protein (Fragment) | -2.51 |  |
| 264271 | *4th Best Hit ACC No.: NP_199811 Arabidopsis thaliana* | 4th Best Hit:ATP binding / kinase/ protein kinase/ protein serine/threonine kinase/ protein-tyrosine kinase | -2.42 |  |
| 261255 | *Danio rerio* | PREDICTED: similar to ADP-ribosylation factor guanine nucleotide-exchange factor 2 | -2.32 |  |
| 21108 | *Aplysia californica* | PKG | -2.30 |  |
| 17095 | *Phytophthora infestans* | putative nuclear LIM factor interactor-interacting protein spore-specific form | -2.08 |  |
| 37727 | *Strongylocentrotus purpuratus* | PREDICTED: similar to calcium/calmodulin-dependent protein kinase 1D | -2.03 |  |
| 12403 | *Chlamydomonas reinhardtii* | chromatin-remodelling complex ATPase ISWI2 | -2.02 |  |
| **Regulation of transcription** | |  |  |  |
| 24112 | *-* | - | +20.56 |  |
| 8292 | *Dictyostelium discoideum* | hypothetical protein DDB0188772 | +8.79 |  |
| 23658 | *Danio rerio* | putative HRP2 transcription factor | +3.50 |  |
| 268655 | *Cryptosporidium hominis TU502* | hypothetical protein Chro.80061 | +3.36 |  |
| 6389 | *Strongylocentrotus purpuratus* | PREDICTED: similar to Programmed cell death protein 2 (Zinc finger protein Rp-8) | +3.10 |  |
| 7788 | - | Hypothetical Protein No BLAST result: ProSite- F-box domain profile | +2.56 |  |
| 262598 | *Strongylocentrotus purpuratus* | PREDICTED: similar to polymerase II | +2.34 |  |
| 23922 | *Arabidopsis thaliana* | DNA binding / transcription factor | +2.31 |  |
| 263046 | *Chlamydia muridarum Nigg* | helicase, putative | +2.05 |  |
| 27451 | *Canis familiaris* | PREDICTED: similar to RuvB-like 2 (48-kDa TATA box-binding protein-interacting protein) | +2.01 |  |
| 10766 | *4th Best Hit ACC No.: XP_214668 Rattus norvegicus* | 4th Best Hit:PREDICTED: similar to transcription factor HSF4b isoform | -9.90 |  |
| 25890 | *Neurospora crassa OR74A* | hypothetical protein | -4.48 |  |
| 264494 | *Strongylocentrotus purpuratus* | PREDICTED: similar to sirtuin (silent mating type information regulation 2 homolog) 6 | -4.05 |  |
| 22372 | *Anopheles gambiae str. PEST* | ENSANGP00000019772 | -3.90 |  |
| 9688 | *Dictyostelium discoideum* | putative basic-leucine zipper transcription factor | -3.56 |  |
| 25332 | *-* | - | -3.36 |  |
| 23665 | *Oncorhynchus mykiss* | heat shock transcription factor 1 isoform b | -3.16 |  |
| 8497 | *Drosophila pseudoobscura* | GA10981-PA | -2.99 |  |
| 23411 | *4th Best Hit ACC No.: AAL90065 Drosophila melanogaster* | 4th Best Hit:AT13703p | -2.56 |  |
| 9571 | *Arabidopsis thaliana* | AT-HSFC1; DNA binding / transcription factor | -2.50 |  |
| 25782 | *Homo sapiens* | splicing coactivator subunit SRm300 | -2.48 |  |
| 1785 | *Arabidopsis thaliana* | Athsf1: heat shock factor | -2.13 |  |
| 5913 | *Dictyostelium discoideum* | hypothetical protein DDB0218102 | -2.12 |  |
| **Methylation** | | | | |
| 20797 | *delta proteobacterium MLMS-1* | putative sarcosine-dimethylglycine methyltransferase | +7.99 |  |
| 21929 | *Synechococcus sp. CC9605* | putative RNA methylase family UPF0020 | +7.81 |  |
| 264008 | *Bacillus cereus subsp. cytotoxis NVH 391-98* | Macrocin-O-methyltransferase | +7.43 | (+2.25 Si; +3.53 Fe; +2.12 pH)^b^ |
| 30915 | *Pseudomonas syringae pv. syringae B728a* | ribosomal protein L11 methyltransferase | +4.72 |  |
| 269942 | *Arabidopsis thaliana* | SHM1 (SERINE HYDROXYMETHYLTRANSFERASE 1); glycine hydroxymethyltransferase | +3.77 | (-2.92 Si)^b^ |
| 2032 | - | Hypothetical Proteins No BLAST results-PFAM: Methyltransferase domain | +3.70 |  |
| 9966 | *Pelodictyon phaeoclathratiforme BU-1* | Methyltransferase FkbM | +2.69 |  |
| 28300 | *Apis mellifera* | PREDICTED: similar to ENSANGP00000016944 | +2.44 |  |
| 9661 | - | Hypothetical Protein No BLAST result; PFAM: Methyltransferase small domain | -2.33 |  |
| **Ubiquitin proteasome system** | | | | |
| 11063 |  | Hypothetical Protein No BLAST result; PFAM: Proteasome A-type and B-type | +6.23 |  |
| 37861 | *Schizosaccharomyces pombe 972h-* | hypothetical protein SPBC119.01 | +4.41 |  |
| 21126 | - | Hypothetical Protein No BLAST result; PFAM: Ubiquitin-conjugating enzyme | +3.88 |  |
| 7601 | *Plasmodium falciparum 3D7* | ubiquitin carboxyl-terminal hydrolase, putative | +3.51 |  |
| 31658 | *3nd Hit: ACC No. BAE00887 Macaca fascicularis* | 3nd Hit:unnamed protein product | +3.40 |  |
| 40483 | *Phytophthora infestans* | proteasome subunit | +3.24 |  |
| 269593 | *Anopheles gambiae str. PEST* | ENSANGP00000017473 | +3.17 |  |
| 33649 | *Aegilops tauschii* | beta1 proteasome-7D | +3.02 |  |
| 7964 | *Zea mays* | putative beta 4 proteasome subunit | +3.02 |  |
| 22097 | *Dictyostelium discoideum* | hypothetical protein DDB0216685 | +2.93 |  |
| 33725 | *Medicago truncatula* | Mov34-1; Flagellar motor switch protein FliG-like | +2.87 |  |
| 28028 | *3nd Hit: ACC No. AAP86661 Arabidopsis thaliana* | 3nd Hit:26S proteasome subunit RPN6a | +2.70 |  |
| 39157 | *Brassica napus* | 26S proteasome subunit 4-like protein | +2.54 |  |
| 267929 | *unidentified* | Skp1-like protein | +2.44 |  |
| 34018 | *Strongylocentrotus purpuratus* | PREDICTED: similar to 26S proteasome non-ATPase regulatory subunit 6 | +2.32 |  |
| 39802 | *Tetraodon nigroviridis* | unnamed protein product | +2.31 |  |
| 1845 | *Arabidopsis thaliana* | AT5G45620: 26S proteasome regulatory subunit, putative (RPN9) | +2.19 |  |
| 26393 | *Avicennia marina* | ubiquitin conjugating protein | +2.08 |  |
| 10336 | *Strongylocentrotus purpuratus* | PREDICTED: similar to F-box only protein 31 | -3.70 |  |
| 35107 | *Apis mellifera* | PREDICTED: similar to ubiquitin-conjugating enzyme E2, J1 | -2.03 |  |
| **Protein catabolism** | | | | |
| 1747 | *Tribolium castaneum* | LOC660463: similar to calpain, small subunit 1 | +11.55 |  |
| 6809 | *-* | Hypothetical Protein No BLAST result | +11.10 |  |
| 14899 | *Arabidopsis thaliana* | DegP protease precursor | +6.14 |  |
| 1788 | - | Hypothetical Proteins No BLAST results-Prosite: Serine proteases, subtilase family, aspartic acid active site | +6.04 |  |
| 38121 | *Oceanicaulis alexandrii HTCC2633* | endopeptidase Clp: ATP-binding subunit B, clpB | +3.66 |  |
| 20993 | *Magnetospirillum magneticum AMB-1* | ATP-dependent protease HslVU | +3.34 |  |
| 269541 | *3nd Hit: ACC No. CAG32961 Populus alba x Populus tremula* | 3nd Hit:putative auxin-amidohydrolase precursor | +3.02 | (+2.79 pH)^b^ |
| 264293 | *Oryza sativa (japonica cultivar-group)* | putative DegP2 protease | +3.01 |  |
| 34684 | *Crocosphaera watsonii WH 8501* | Peptidase S1, chymotrypsin:PDZ/DHR/GLGF | +2.99 |  |
| 26436 | *Arabidopsis thaliana* | metalloendopeptidase | +2.92 |  |
| 24560 | *Canis familiaris* | PREDICTED: similar to Protein disulfide-isomerase A4 precursor (Protein ERp-72) (ERp72) isoform 3 | +2.79 |  |
| 261093 | *Oceanospirillum sp. MED92* | peptidase, U7 family protein | +2.72 |  |
| 22011 | *Blastocladiella emersonii* | mitochondrial processing peptidase alpha subunit | +2.59 |  |
| 262525 | *Branchiostoma floridae* | 3rd Best Hit: LMP7-like protein | +2.53 |  |
| 2352 | *Plasmodium falciparum 3D7* | hypothetical protein [Plasmodium falciparum 3D7] | +2.41 |  |
| 2674 | - | Hypothetical Proteins No BLAST results-PFAM: Reprolysin (M12B) family zinc metalloprotease | +2.38 |  |
| 24295 | *Medicago truncatula* | Proteasome/cyclosome, regulatory subunit | +2.36 |  |
| 37338 | *Emiliania huxleyi* | putative protein disulfide isomerase precursor | +2.25 |  |
| 1366 | *Theileria annulata strain Ankara* | TA18155: protease | +2.22 |  |
| 36310 | *3nd Hit: ACC No. XP_882178 Bos taurus* | 3nd Hit:PREDICTED: similar to CAAX prenyl protease 1 homolog | +2.19 |  |
| 41169 | *Cucumis melo* | mitochondrial processing peptidase beta subunit | +2.13 |  |
| 18613 | *Synechococcus sp. JA-2-3B'a(2-13)* | ATP-dependent Clp protease, proteolytic subunit ClpP | +2.01 |  |
| 21333 | *Hahella chejuensis KCTC 2396* | PPE-repeat protein | -3.72 |  |
| 23419 | *Streptomyces avermitilis MA-4680* | protease | -3.02 |  |
| 260832 | *Synechocystis sp. PCC 6803* | leader peptidase I | -2.62 |  |
| 32971 | *Chaetoceros compressum* | HI-5a | -2.55 |  |
| 268220 | *Clostridium acetobutylicum ATCC 824* | Secreted protease metal-dependent protease | -2.47 |  |
| 264337 | *Oceanobacillus iheyensis HTE831* | hypothetical conserved protein | -2.23 |  |
| **Chaperones/Heat-shock response** | | | | |
| 261702 | *Capsicum frutescens* | chloroplast small heat shock protein class I | +146.71 |  |
| 6793 | *Bdellovibrio bacteriovorus HD100* | low molecular weight heat shock protein | +16.69 | (-2.2 Fe)^c^ |
| 37367 | *3nd Hit: ACC No. YP_381197 Synechococcus sp. CC9605* | 3nd Hit:Peptidylprolyl isomerase | +12.19 |  |
| 2720 | *Ashbya gossypii ATCC 10895* | AGOS_ADL388W: ADL388Wp | +7.87 | (-2.8 Fe)^c^ |
| 263422 | *Phaeodactylum tricornutum* | BiP | +6.55 |  |
| 38191 | *Prunus dulcis* | heat shock protein 60 | +5.47 |  |
| 34810 | *Hordeum vulgare subsp. vulgare* | CRH1: calreticulin | +5.26 |  |
| 269240 | *Parvularcula bermudensis HTCC2503* | molecular chaperone DnaK | +4.67 |  |
| 29506 | *3nd Hit: ACC No. NP_563961 Arabidopsis thaliana* | 3nd Hit:CPN10 (CHAPERONIN 10) | +4.31 |  |
| 21965 | *Emiliania huxleyi* | putative protein disulfide isomerase precursor | +4.14 |  |
| 24932 | *Emiliania huxleyi* | putative protein disulfide isomerase precursor | +3.94 |  |
| 25599 | *Gallus gallus* | PREDICTED: similar to endoplasmic reticulum chaperone SIL1, homolog of yeast; BiP-associated protein | +3.85 |  |
| 22766 | *Toxoplasma gondii* | HSP90-like protein | +2.63 |  |
| 33153 | *Arabidopsis thaliana* | peptidyl-prolyl cis-trans isomerase | +2.56 |  |
| 268062 | *4th Best Hit ACC No.: YP_479117 Synechococcus sp. JA-2-3B'a(2-13)* | 4th Best Hit:chaperone protein DnaJ | +2.48 |  |
| 21734 | *Cryptosporidium hominis TU502* | hypothetical protein Chro.80380; Prosite: DnaJ-1 | +2.26 |  |
| 11286 | *4th Best Hit ACC No.: NP_173585 Arabidopsis thaliana* | 4th Best Hit:unknown protein; PFAM: DnaJ domain | +2.16 |  |
| 2678 | *Geobacillus kaustophilus HTA426* | GK0656: post-translocation molecular chaperone | +2.16 |  |
| 2211 | - | Hypothetical Proteins No BLAST results-Prosite: FKBP-type peptidyl-prolyl cis-trans isomerase domain | +2.08 |  |
| 20186 | *Acidobacteria bacterium Ellin345* | Chaperone DnaJ | -3.87 | (+4.68 N)^b^ |
| 1170 | - | Hypothetical Proteins No BLAST results-PFAM: HSF-type DNA-binding | -2.31 |  |
| **Metabolism** | | | | |
| 21141 | *Colwellia psychrerythraea 34H* | riboflavin biosynthesis protein RibD | +16.31 |  |
| 25797 | *Dictyostelium discoideum* | hypothetical protein DDB0185465; PFAM: Helix-loop-helix DNA-binding domain | +14.94 | (+2.58 T)^b^ |
| 267987 | *Isochrysis galbana* | chloroplast O-acetyl-serine lyase | +13.10 |  |
| 261750 | *5th Best Hit ACC No.: AAU84235 uncultured archaeon GZfos3D4* | 5th Best Hit: putative glycerate kinase | +11.14 |  |
| 7886 | - | Hypothetical Protein No BLAST result: ProSite- Gcn5-related N-acetyltransferase (GNAT) domain profil | +10.92 |  |
| 25392 | *4th Best Hit ACC No.: ZP_00051562 Magnetospirillum magnetotacticum MS-1* | 4th Best Hit:COG2931: RTX toxins and related Ca2+-binding proteins | +8.91 |  |
| 22030 | *Caenorhabditis briggsae* | Hypothetical protein CBG16864; 2nd HIT: phosphoglycolate phosphatase | +8.65 | (+4.4 Si; +3.02 Fe)^b^ |
| 6209 | *Arabidopsis thaliana* | aminocarboxymuconate-semialdehyde decarboxylase/ hydrolase | +8.06 |  |
| 29782 | *Xenopus tropicalis* | Integrin beta 4 binding protein | +7.38 |  |
| 10468 | *Listeria innocua* | lin0343 | +7.01 |  |
| 8860 | - | Hypothetical Protein No BLAST result; PFAM: Glutathione S-transferase | +6.05 |  |
| 7540 | *Arabidopsis thaliana* | catalytic | +6.03 |  |
| 10628 | - | Hypothetical Protein No BLAST result: ProSite- Isopenicillin N synthetase signature 1 | +5.87 |  |
| 8303 | - | Hypothetical Protein No BLAST result: ProSite- Ribosomal protein S16 signature | +5.80 |  |
| 31785 | *3nd Hit: ACC No. BAD54224 Oryza sativa (japonica cultivar-group)* | 3nd Hit:putative 4-methyl-5(B-hydroxyethyl)-thiazol monophosphate biosynthesis enzyme | +5.40 |  |
| 17214 | *Sphingomonas sp. SKA58* | acetylglutamate kinase | +5.19 |  |
| 38294 | *Saccharomyces cerevisiae* | Putative cysteine synthase, localized to the mitochondrial outer membrane; Ygr012wp | +5.13 |  |
| 10612 | *Agrobacterium tumefaciens str. C58* | thiosulfate sulfurtransferase | +4.57 |  |
| 5852 | *3nd Hit: ACC No. ZP_00109643 Nostoc punctiforme PCC 73102* | 3nd Hit:COG0625: Glutathione S-transferase | +4.38 |  |
| 24965 | *Oryza sativa (japonica cultivar-group)* | MAPEG family, putative | +3.97 |  |
| 6683 | *Caenorhabditis elegans* | Hypothetical protein F47A4.2 | +3.63 |  |
| 34340 | *Arabidopsis thaliana* | Gene ontology: electron transport | +3.59 |  |
| 22874 | *Leishmania major strain Friedlin* | proteophosphoglycan 5 | +3.54 |  |
| 18220 | *Volvox carteri* | GTP-binding protein | +3.43 |  |
| 264268 | *Nostoc punctiforme PCC 73102* | COG2197: Response regulator containing a CheY-like receiver domain and an HTH DNA-binding domain | +3.32 |  |
| 30659 | *Silicibacter pomeroyi DSS-3* | metallo-beta-lactamase family protein | +2.98 |  |
| 22368 | *Oryza sativa (japonica cultivar-group)* | OSJNBa0081L15.19 | +2.73 |  |
| 31543 | *Oryza sativa (japonica cultivar-group)* | ATPase like | +2.68 |  |
| 1615 | *Leishmania major strain Friedlin* | LMJ_0986: proteophosphoglycan 5 | +2.68 |  |
| 24017 | *-* | PFAM: Calcineurin-like phosphoesterase | +2.58 | (+2.37 Si) ^b^ |
| 13572 | *Cryptosporidium hominis TU502* | methionine -- tRNA ligase | +2.46 |  |
| 38651 | *Tetraodon nigroviridis* | unnamed protein product | +2.38 |  |
| 20998 | *Xenopus laevis* | MGC80292 protein | +2.26 |  |
| 18099 | *Apis mellifera* | PREDICTED: similar to CG32857-PA | +2.20 |  |
| 9118 | *Psychrobacter sp. PRwf-1* | Molybdopterin cofactor biosynthesis MoaC region | +2.14 |  |
| 1302 | *Propionibacterium acnes KPA171202* | PPA0809: putative phosphoglycerate mutase/fructose-2,6-bisphosphatase | +2.03 |  |
| 7417 | *-* | Pattern: ATP synthase alpha beta subunit | -6.74 | (-8.4 Fe)^c^ |
| 5371 | *Chlamydomonas reinhardtii* | p-hydroxyphenylpyruvate dioxigenase | -5.41 |  |
| 5769 | *Dictyostelium discoideum* | hypothetical protein DDB0205801 | -5.19 |  |
| 38359 | *Plasmodium yoelii yoelii str. 17XNL* | glutamate dehydrogenase | -5.09 |  |
| 268335 | *Desulfovibrio desulfuricans G20* | Glu/Leu/Phe/Val dehydrogenase family protein | -4.07 |  |
| 2865 | *Leishmania major strain Friedlin* | LMJ_0986: proteophosphoglycan 5 | -4.00 |  |
| 264582 | *3nd Hit: ACC No. BAC45252 Nocardiopsis prasina* | 3nd Hit:family19 chitinase | -3.99 |  |
| 263096 | *Medicago truncatula* | Glycoside hydrolase, family 19; Chitin-binding, type 1 | -3.74 |  |
| 24521 | *Dictyostelium discoideum* | hypothetical protein DDB0187034; PFAM: Glutamine amidotransferases class-II | -3.59 | (+2.16 pH) ^b^ |
| 269264 | *Drosophila melanogaster* | GRAAL2 protein | -3.34 |  |
| 22237 | *Vibrio fischeri ES114* | chitodextrinase precursor | -3.29 |  |
| 35532 | *Alteromonas macleodii 'Deep ecotype'* | UDP-glucose 4-epimerase | -3.22 |  |
| 6568 | *Giardia lamblia ATCC 50803* | hypothetical protein GLP_587_119251_118319; PFAM: Aminotransferase class IV | -3.19 |  |
| 42123 | *Dictyostelium discoideum* | hypothetical protein DDB0187034; PFAM: Glutamine amidotransferases class-II | -3.10 | (-2.49 T; +2.32 pH) ^b^ |
| 22855 | *Leishmania major strain Friedlin* | proteophosphoglycan 5 | -3.01 |  |
| 21774 | *Caenorhabditis briggsae* | Hypothetical protein CBG23547 | -3.01 |  |
| 9163 | *Anopheles gambiae str. PEST* | ENSANGP00000011668 | -2.87 |  |
| 21439 | *Dictyostelium discoideum* | hypothetical protein DDB0205801; PFAM:  PT repeat | -2.86 |  |
| 262153 | *Aspergillus nidulans FGSC A4* | hypothetical protein AN5077.2; PFAM:  Glycosyl hydrolases family 18 | -2.83 | (-3.16 T)^b^ |
| 8952 | *Dictyostelium discoideum* | hypothetical protein DDB0187034; PFAM:  Glutamine amidotransferases class-II- | -2.80 | (-3.83 Si)^b^ |
| 23700 | *Burkholderia gladioli* | chitinase B | -2.78 |  |
| 12024 | - | Hypothetical Protein No BLAST result: ProSite- EF-hand calcium-binding domain | -2.74 |  |
| 268366 | *Halobacterium sp. NRC-1* | chitinase; Chi | -2.74 |  |
| 11168 | *Dictyostelium discoideum* | hypothetical protein DDB0167328 | -2.60 |  |
| 23376 | *Leishmania major strain Friedlin* | proteophosphoglycan ppg4 | -2.59 |  |
| 31108 | *Ginkgo biloba* | trehalose-6-phosphate synthase | -2.48 |  |
| 268966 | *Thermococcus kodakarensis KOD1* | chitinase, containing dual catalytic domains | -2.48 |  |
| 268234 | *Rhodobacterales bacterium HTCC2654* | putative RTX toxin | -2.45 |  |
| 5687 | *Geobacter sulfurreducens PCA* | cobyrinic acid a,c-diamide synthase family protein | -2.35 |  |
| 30986 | *Arabidopsis thaliana* | putative SCO1 protein | -2.33 |  |
| 24078 | *Zea mays* | Hox2a | -2.31 |  |
| 23303 | *-* | Hypothetical Protein No BLAST result | -2.28 |  |
| 4740 | *Leishmania major strain Friedlin* | proteophosphoglycan 5 | -2.24 |  |
| 14719 | *3nd Hit: ACC No. NP_001031709 Arabidopsis thaliana* | 3nd Hit:unknown protein | -2.22 |  |
| 22624 | *Staphylococcus haemolyticus JCSC1435* | unnamed protein product; Pattern: ATP synthase alpha beta subunit | -2.18 |  |
| 20732 | *Anopheles gambiae str. PEST* | ENSANGP00000010886 | -2.15 |  |
| 5035 | *Streptomyces coelicolor A3(2)* | hypothetical protein SCO2299 | -2.12 |  |
| 260939 | *Tetraodon nigroviridis* | unnamed protein product | -2.10 |  |
| 37493 | *Methylobacillus flagellatus KT* | formyltetrahydrofolate deformylase | -2.07 |  |
| 17193 | *Arabidopsis thaliana* | catalytic | -2.06 |  |
| **Silicon metabolism** | | | | |
| 268895 | - | *-* | -14.94 | (+7.15 Si)^b^ |
| 22708 | *Thalassiosira pseudonana* | silaffin precursor | -2.734 |  |
| **Lipid metabolism** | | | | |
| 1909 | Hypothetical Protein No BLAST result | Hypothetical Proteins No BLAST results-PFAM: Phytanoyl-CoA dioxygenase (PhyH) 29897.8kDa | +13.32 | (+2.09 T)^b^; (+6.8 Fe)^c^ |
| 22993 | *Pseudoalteromonas tunicata D2* | acetyl-CoA carboxylase | +9.77 | (+2.87 Si; +3 Fe) ^b^ |
| 263053 | *Arabidopsis thaliana* | SEC14-like protein | +5.98 |  |
| 23858 | *Rhodospirillum rubrum ATCC 11170* | Glycerophosphoryl diester phosphodiesterase | +5.78 |  |
| 29867 | *Thalassiosira pseudonana* | long chain acyl-coA synthetase | +4.18 |  |
| 11486 | - | Hypothetical Protein No BLAST result; PFAM: Phytanoyl-CoA dioxygenase (PhyH) | +4.00 |  |
| 4704 | *Cryptococcus neoformans var. neoformans JEC21* | glycerol-3-phosphate O-acyltransferase, putative | +2.84 |  |
| 40073 | *3nd Hit: ACC No. NP_565939 Arabidopsis thaliana* | 3nd Hit:SFD1 (Suppressor of fatty acid desaturase deficiency 1) | +2.76 |  |
| 4270 |  | PFAM:scramblase | +2.25 |  |
| 20846 | *Medicago truncatula* | Diacylglycerol acyltransferase | +2.19 |  |
| 33895 | *Suberites domuncula* | dihydroxyacetonephosphate synthase | +2.02 |  |
| 269328 | *Rhodopseudomonas palustris HaA2* | Propionyl-CoA carboxylase | -6.25 |  |
| 32789 | *Magnetospirillum magnetotacticum MS-1* | COG4799: Acetyl-CoA carboxylase, carboxyltransferase component (subunits alpha and beta) | -3.01 |  |
| 26365 | *Xenopus tropicalis* | Hydroxyacyl-Coenzyme A dehydrogenase/3-ketoacyl-Coenzyme A thiolase/enoyl-Coenzyme A hydratase (trifunctional protein), alpha subunit | -2.43 | (+2.18 Si; -3.32 T) ^b^ |
| 3244 | *Apis mellifera* | LOC409614: similar to GXIVsPLA2 CG17035-PA, isoform A | -2.11 |  |
|  |  |  |  |  |
| **Carbon fixation** | | | | |
| 25116 | *Apis mellifera* | PREDICTED: similar to GA16139-PA | +10.81 |  |
| 42577 | *Phaeodactylum tricornutum* | phosphoglycerate kinase precursor | +3.27 | (-2.28 Si)^b^ |
| 20731 | *Drosophila melanogaster* | CG1907-PA  Oxoglutarate/malate carrier protein | +3.15 | (+2.73 T)^b^ |
| 261823 | *Oryza sativa (indica cultivar-group)* | sedoheptulose-1,7-bisphosphatase precursor | +2.13 |  |
| 264583 | *Vibrio cholerae O1 biovar eltor str. N16961* | pyruvate kinase I | -2.85 |  |
| **Transport** |  |  |  |  |
| 11484 | *Xenopus laevis* | potassium channel xKv4.3 | +24.54 |  |
| 263118 | *3nd Hit: ACC No. ZP_01083654 Synechococcus sp. WH 5701* | 3nd Hit:ABC transporter, ATP binding component, possibly for oligopeptides | +8.49 |  |
| 5476 | *Tribolium castaneum* | PREDICTED: similar to solute carrier family 25 (mitochondrial carrier, palmitoylcarnitine transporter), member 29 (predicted) | +8.32 |  |
| 9705 | *Tetraodon nigroviridis* | unnamed protein product | +6.80 |  |
| 1835 | *Aspergillus oryzae* | AO090003000997: - | +4.38 |  |
| 260789 | *Hahella chejuensis KCTC 2396* | predicted Co/Zn/Cd cation transporters | +3.84 |  |
| 22186 | *3nd Hit: ACC No. YP_604963 Deinococcus geothermalis DSM 11300* | 3nd Hit:major facilitator superfamily MFS_1 | +3.17 |  |
| 3649 | *Staphylococcus haemolyticus JCSC1435* | SH0040: hypothetical protein | +2.73 |  |
| 31928 | *Bordetella bronchiseptica RB50* | ABC transporter, ATP-binding protein | +2.69 |  |
| 262743 | *Tribolium castaneum* | PREDICTED: similar to choline transporter-like protein 2 | +2.63 |  |
| 261431 | *Cryptococcus neoformans var. neoformans JEC21* | ATP-binding cassette (ABC) transporter, putative | +2.60 |  |
| 7597 | *Strongylocentrotus purpuratus* | PREDICTED: similar to membrane transporter like (50.3 kD) (2E985) | +2.59 |  |
| 262136 | *Candida albicans SC5314* | hypothetical protein CaO19_5444 | +2.42 |  |
| 6592 | *Rhodopirellula baltica SH 1* | probable ABC transporter (substrate-binding protein) | +2.33 |  |
| 1704 | - | Hypothetical Protein No BLAST result | +2.31 |  |
| 264807 | *Pagrus major* | ATPase H+ transporting lysosomal vacuolar proton pump | +2.27 | (-2.4 Si)^b^ |
| 8159 | *Cryptosporidium hominis TU502* | myosin heavy chain | +2.14 |  |
| 19639 | *Debaryomyces hansenii CBS767* | hypothetical protein DEHA0G21813g | +2.10 |  |
| 8613 | *Plasmodium falciparum 3D7* | snrnp protein, putative | +2.08 |  |
| 31011 | *Debaryomyces hansenii CBS767* | hypothetical protein DEHA0F12925g | -5.25 |  |
| 23027 | *Leishmania major strain Friedlin* | proteophosphoglycan 5 | -3.01 |  |
| 7123 | *Cryptococcus neoformans var. neoformans JEC21* | MipD, putative | -2.94 |  |
| 7974 | *Cryptosporidium hominis TU502* | myosin heavy chain | -2.69 | (-2.87 T)^b^ |
| 21585 | *Mus musculus* | unnamed protein product | -2.62 |  |
| 22783 | *Candida albicans SC5314* | hypothetical protein CaO19.7095 | -2.28 |  |
| 4507 | *Strongylocentrotus purpuratus* | PREDICTED: similar to zinc transporter ZTL1 isoform 1 | -2.18 |  |
| 20952 | *Vibrio vulnificus CMCP6* | Small-conductance mechanosensitive channel | -2.13 |  |
| 24796 | *Cryptococcus neoformans var. neoformans JEC21* | vacuolar protein sorting 41, putative | -2.09 |  |
| 25062 | *Pisum sativum* | Vacuolar sorting receptor 1 precursor (BP-80) | -2.05 |  |
| 22668 | *3nd Hit: ACC No. ZP_01015351 Rhodobacterales bacterium HTCC2654* | 3nd Hit:hypothetical protein RB2654_17991 | -2.04 |  |
| **Unassigned** | | | | |
| 264384 | *Thalassiosira pseudonana* | Cue7 | +37.18 | (+3 pH)^b^; (-14.3 Fe)^c^; (Cu)^d^ |
| 8240 | *-* | Hypothetical Protein No BLAST result | +26.11 |  |
| 9099 | *-* | Hypothetical Protein No BLAST result | +23.22 |  |
| 8988 | - | Hypothetical Protein No BLAST result; PFAM: Prolyl oligopeptidase family | +19.10 |  |
| 25650 | *-* | Hypothetical Protein No BLAST result | +19.04 |  |
| 22860 | *-* | Hypothetical Protein No BLAST result | +17.41 | (-2.03 Si)^b^ |
| 25127 | *Homo sapiens* | Transmembrane protein 43 | +17.13 |  |
| 7672 | *3nd Hit: ACC No. YP_478184 Synechococcus sp. JA-2-3B'a(2-13)* | 3nd Hit:hypothetical protein CYB_1972 | +15.64 |  |
| 21831 | - | Hypothetical Protein No BLAST result; PFAM: 2OG-Fe(II) oxygenase superfamily | +15.39 |  |
| 269876 | *Thalassiosira pseudonana* | Cue6 | +14.90 | (-20.9 Fe)^c^; (Cu)^d^ |
| 11562 | *Leishmania major strain Friedlin* | proteophosphoglycan 5 | +14.81 |  |
| 10867 | *Arabidopsis thaliana* | unknown protein | +14.38 |  |
| 5128 | *Trypanosoma cruzi strain CL Brener* | TPR-repeat protein | +14.28 |  |
| 10416 | *Dictyostelium discoideum* | hypothetical protein DDB0218051 | +13.29 |  |
| 11488 | *Danio rerio* | Hypothetical protein LOC550429 | +12.01 |  |
| 7336 | *-* | Hypothetical Protein No BLAST result | +11.91 |  |
| 23620 | *Spodoptera exigua nucleopolyhedrovirus* | ORF4 hoar | +10.83 | (+5.16 pH)^b^(-14.3 Fe)^c^ |
| 8903 | *Schistosoma japonicum* | SJCHGC00943 protein | +9.92 |  |
| 7337 | *-* | Hypothetical Protein No BLAST result | +9.81 |  |
| 23393 | - | Hypothetical Protein No BLAST result; Peptidase family S51 | +9.30 |  |
| 23706 | - | Hypothetical Protein No BLAST result; PFAM: Family of unknown function (DUF500) | +9.27 |  |
| 25915 | *-* | Hypothetical Protein No BLAST result | +8.95 |  |
| 22312 | *3nd Hit: ACC No. XP_235927 Rattus norvegicus* | 3nd Hit:PREDICTED: similar to hypothetical protein MGC20983 | +8.80 |  |
| 25353 | - | Hypothetical Protein No BLAST result; PFAM: eubacterial secY protein | +8.71 |  |
| 5409 | - | Hypothetical Protein No BLAST result; PFAM: HEAT repeat | +8.61 |  |
| 35031 | *Caenorhabditis elegans* | Hypothetical protein F56C11.3 | +8.53 |  |
| 1926 | - | Hypothetical Proteins No BLAST results-PFAM: GTP1/OBG 28497.9kDa | +8.45 |  |
| 7554 | *Danio rerio* | PREDICTED: similar to mKIAA1626 protein | +8.41 |  |
| 25441 | *Candida glabrata* | unnamed protein product | +8.37 |  |
| 3976 | - | Hypothetical Proteins No BLAST results-PFAM: Helicase associated domain | +8.12 | (+3.22 Si; +2.71 Fe)^b^ |
| 25360 | *Caenorhabditis briggsae* | Hypothetical protein CBG20540 | +8.06 |  |
| 9345 | *-* | Hypothetical Protein No BLAST result | +7.86 |  |
| 3765 | - | Hypothetical Proteins No BLAST results-PFAM: Tetratricopeptide repeat | +7.67 |  |
| 11569 | *-* | Hypothetical Protein No BLAST result | +7.51 |  |
| 32459 | *Synechococcus elongatus PCC 7942* | membrane-associated 30 kD protein-like | +7.36 |  |
| 11698 | - | Hypothetical Protein No BLAST result; PFAM: START domain | +7.18 |  |
| 3422 | - | Hypothetical Proteins No BLAST results-PFAM: Globin | +7.18 |  |
| 262540 | *3nd Hit: ACC No. NP_870856 Rhodopirellula baltica SH 1* | 3nd Hit:conserved hypothetical protein-putative cobalamin synthesis protein CobW | +7.07 |  |
| 21710 | *Dictyostelium discoideum* | hypothetical protein DDB0188495 | +6.92 |  |
| 10434 | *-* | - | +6.68 |  |
| 33437 | *Sulfitobacter sp. NAS-14.1* | endoribonuclease L-PSP family protein | +6.62 |  |
| 264339 | *Gossypium barbadense* | fiber protein Fb38 | +6.47 |  |
| 20597 | - | Hypothetical Protein No BLAST result; PFAM: Protein of unknown function (DUF1350) | +6.24 |  |
| 268596 | *3nd Hit: ACC No. AAH07893 Homo sapiens* | 3nd Hit:NOC4L protein | +6.24 |  |
| 6979 | *3nd Hit: ACC No. YP_066036 Desulfotalea psychrophila LSv54* | 3nd Hit:related to NonF protein | +6.16 |  |
| 22886 | *Arabidopsis thaliana* | unknown protein | +6.07 |  |
| 35401 | *Klebsiella pneumoniae* | CobW | +5.82 |  |
| 7419 | *3nd Hit: ACC No. Q5R6K5 Pongo pygmaeus* | 3nd Hit:hypothetical protein | +5.79 |  |
| 6633 | *Plasmodium yoelii yoelii str. 17XNL* | hypothetical protein PY00604 | +5.75 |  |
| 7999 | - | Hypothetical Protein No BLAST result; PFAM: TLD | +5.73 |  |
| 5675 | - | Hypothetical Protein No BLAST result; PFAM: PPR repeat | +5.53 |  |
| 11747 | - | Hypothetical Protein No BLAST result | +5.39 |  |
| 40489 | *Arabidopsis thaliana* | unknown | +5.16 |  |
| 25076 | *Xenopus tropicalis* | hypothetical protein LOC549937 | +4.94 |  |
| 11425 | *-* | Hypothetical Protein No BLAST result | +4.93 |  |
| 24044 | *-* | Hypothetical Protein No BLAST result | +4.75 |  |
| 4372 |  | hypothetical protein | +4.72 |  |
| 21431 | *-* | - | +4.60 |  |
| 11383 | *-* | Hypothetical Protein No BLAST result | +4.57 | (+3.03 pH)^b^ |
| 23850 | *Staphylococcus aureus subsp. aureus USA300* | LPXTG-motif cell wall surface anchor family protein | +4.51 |  |
| 10858 | *Apis mellifera* | PREDICTED: similar to CG18255-PA, isoform A | +4.50 |  |
| 9460 | *3nd Hit: ACC No. XP_746138 Plasmodium chabaudi chabaudi* | 3nd Hit:Plasmodium chabaudi chabaudi kelch protein | +4.32 |  |
| 21519 | *Nostoc sp. PCC 7120* | all5250 | +4.29 | (+2.42 T)^b^ |
| 1555 | *Entamoeba histolytica HM-1:IMSS* | 42.t00003: hypothetical protein | +4.17 |  |
| 21804 | - | Hypothetical Protein No BLAST result; PFAM: Ankyrin repeat | +4.17 |  |
| 262455 | *Desulfovibrio vulgaris subsp. vulgaris str. Hildenborough* | hemolysin III | +4.16 |  |
| 24826 | - | Hypothetical Protein No BLAST result; PFAM: 3'5'-cyclic nucleotide phosphodiesterase | +4.13 |  |
| 9388 | *Plasmodium falciparum 3D7* | hypothetical protein PF11_0049 | +4.12 |  |
| 38991 | *Magnetospirillum magneticum AMB-1* | hypothetical protein amb0594 | +4.01 |  |
| 3650 | - | Hypothetical Proteins No BLAST results-Prosite: Protamine P1 signature, PFAM: Tetratricopeptide repeat | +4.01 |  |
| 8720 | *Tetrahymena thermophila* | nucleolar phosphoprotein | +4.00 |  |
| 24850 | *Caenorhabditis briggsae* | Hypothetical protein CBG24281 | +3.91 |  |
| 24495 | *Gibberella zeae PH-1* | hypothetical protein FG00653.1 | +3.90 |  |
| 7940 | *-* | Hypothetical Protein No BLAST result | +3.88 | (+2.93 T)^b^ |
| 23212 | *Trypanosoma cruzi strain CL Brener* | hypothetical protein | +3.87 |  |
| 6692 | *Oryza sativa (japonica cultivar-group)* | putative spop | +3.79 |  |
| 20737 | *Trypanosoma cruzi strain CL Brener* | hypothetical protein | +3.72 |  |
| 11347 | *-* | Hypothetical Protein No BLAST result | +3.67 |  |
| 25110 | *Dictyostelium discoideum* | hypothetical protein DDB0201756 | +3.65 |  |
| 21911 | *Drosophila pseudoobscura* | GA12224-PA | +3.64 |  |
| 39666 | *3nd Hit: ACC No. YP_320806 Anabaena variabilis ATCC 29413* | 3nd Hit:Cobalamin synthesis protein/P47K | +3.61 |  |
| 11165 | Hypothetical Proteins No BLAST results-PFAM: U1 zinc finger 58547.5kDa | Hypothetical Protein No BLAST result | +3.58 |  |
| 263182 | *3nd Hit: ACC No. YP_478070 Synechococcus sp. JA-2-3B'a(2-13)* | 3nd Hit:hypothetical protein CYB_1852 | +3.48 |  |
| 6473 | *-* | Hypothetical Protein No BLAST result | +3.48 |  |
| 21979 | *Neurospora crassa OR74A* | predicted protein | +3.47 |  |
| 22036 | *Strongylocentrotus purpuratus* | PREDICTED: similar to Zinc phosphodiesterase ELAC protein 2 (Ribonuclease Z 2) (RNase Z 2) (tRNase Z 2) (tRNA 3 endonuclease 2) (ElaC homolog protein 2) | +3.46 |  |
| 4330 | *3nd Hit: ACC No. ZP_01234066 Vibrio angustum S14* | 3nd Hit:hypothetical protein VAS14_14429 | +3.46 |  |
| 5540 | Hypothetical Protein No BLAST result: MW = 37724.3Da | Hypothetical Protein No BLAST result: ProSite- JmjC domain profile; MW = 37724.3 Da | +3.45 |  |
| 4829 | - | Hypothetical Protein No BLAST result; PFAM: Cofilin/tropomyosin-type actin-binding protein | +3.42 |  |
| 12180 | *Dictyostelium discoideum* | hypothetical protein DDB0218648 | +3.36 |  |
| 24718 | *-* | - | +3.33 |  |
| 8353 | *-* | Hypothetical Protein No BLAST result | +3.31 |  |
| 269449 | *Arabidopsis thaliana* | unknown protein | +3.30 |  |
| 1761 | *Arabidopsis thaliana* | AT3G60810: unknown protein | +3.30 | (+2.18 T)^b^ |
| 22443 | *Candida glabrata* | unnamed protein product | +3.27 |  |
| 2895 | - | Hypothetical Proteins No BLAST results-PFAM: Metallo-beta-lactamase superfamily | +3.24 |  |
| 25011 | - | unknown protein | +3.24 |  |
| 22245 | *Arabidopsis thaliana* | unknown protein | +3.17 |  |
| 12189 | - | Hypothetical Protein No BLAST result; PFAM: Extensin-like region | +3.16 |  |
| 268744 | *Dictyostelium discoideum* | hypothetical protein DDB0218132 | +3.13 |  |
| 24529 | *Grouper iridovirus* | unknown protein | +3.11 |  |
| 5452 | *-* | Hypothetical Protein No BLAST result | +3.09 |  |
| 11081 | - | Hypothetical Protein No BLAST result | +3.06 |  |
| 20983 | *Plasmodium falciparum 3D7* | hypothetical protein | +3.03 |  |
| 20927 | *Strongylocentrotus purpuratus* | PREDICTED: similar to transmembrane protein 20 | +2.99 |  |
| 270115 | *Oryza sativa (japonica cultivar-group)* | putative Reg receptor | +2.96 |  |
| 263880 | *Oryza sativa* | methionyl-tRNA synthetase | +2.95 |  |
| 35036 | *5th Best Hit ACC No.: XP_666256 Cryptosporidium hominis TU502* | 5th Best Hit: multi-pass transmembrane protein | +2.95 |  |
| 7160 | - | Hypothetical Proteins No BLAST results- | +2.93 |  |
| 33480 | *Idiomarina loihiensis L2TR* | Uncharacterized conserved protein | +2.93 |  |
| 889 | - | Hypothetical Protein No BLAST result | +2.90 |  |
| 3604 | - | Hypothetical Proteins No BLAST results-PFAM: HIT zinc finger | +2.90 |  |
| 8157 | *Trypanosoma cruzi strain CL Brener* | ADP-ribosylation factor-like protein 3A | +2.89 |  |
| 2200 | - | Hypothetical Proteins No BLAST results-Prosite: Zinc finger RING-type profile | +2.87 |  |
| 21341 | *-* | Hypothetical Protein No BLAST result | +2.85 |  |
| 29705 | *Dictyostelium discoideum* | hypothetical protein DDB0218402 | +2.83 |  |
| 10140 | *Tetraodon nigroviridis* | unnamed protein product | +2.83 |  |
| 21362 | *-* | Hypothetical Protein No BLAST result | +2.82 | (+1.7 N)^b^ |
| 928 | - | Hypothetical Proteins No BLAST results-PFAM: ADP-ribosylglycohydrolase | +2.78 |  |
| 926 | - | Hypothetical Proteins No BLAST results; PFAM: Endoribonuclease L-PSP | +2.72 |  |
| 5241 | *Ustilago maydis 521* | hypothetical protein UM03124.1 | +2.71 |  |
| 10385 | *Frankia sp. EAN1pec* | conserved hypothetical protein | +2.70 | (+3.85 Si; +3.51 Fe)^b^ |
| 8673 | *-* | Hypothetical Protein No BLAST result | +2.66 |  |
| 269487 | *Magnaporthe grisea 70-15* | hypothetical protein MG06157.4 | +2.64 |  |
| 1908 | *Strongylocentrotus purpuratus* | LOC577233: similar to conserved hypothetical protein | +2.64 |  |
| 268300 | - | Hypothetical Protein No BLAST result; PFAM: Anthranilate synthase component I | +2.63 |  |
| 25163 | *-* | Hypothetical Protein No BLAST result | +2.60 |  |
| 9130 | - | Hypothetical Protein No BLAST result; SCP-like extracellular protein | +2.60 |  |
| 2244 | - | Hypothetical Proteins No BLAST results; PFAM: Sjogren's syndrome/scleroderma autoantigen 1 (Autoantigen p27) | +2.58 |  |
| 22978 | *Xenopus laevis* | MGC80226 protein | +2.57 |  |
| 11606 | *Arabidopsis thaliana* | unknown protein | +2.56 |  |
| 269559 | *Candida glabrata* | unnamed protein product | +2.54 |  |
| 23126 | - | Hypothetical Protein No BLAST result; PFAM: TLD | +2.51 |  |
| 3280 | - | Hypothetical Proteins No BLAST results-PFAM: Eukaryotic membrane protein (cytomegalovirus gH-receptor) family | +2.50 |  |
| 2605 | *Tribolium castaneum* | LOC657022: similar to CG18304-PA | +2.45 |  |
| 23929 | *Flavobacterium johnsoniae UW101* | hypothetical protein FjohDRAFT_3304 | +2.45 |  |
| 23969 | *Caenorhabditis briggsae* | Hypothetical protein CBG06865 | +2.43 |  |
| 22547 | *Cryptosporidium hominis TU502* | hypothetical protein Chro.10068 | +2.36 |  |
| 21061 | *Anopheles gambiae str. PEST* | ENSANGP00000022061 | +2.34 |  |
| 31674 | *-* | - | +2.33 |  |
| 268172 | *Arabidopsis thaliana* | unknown protein | +2.30 |  |
| 2996 | - | Hypothetical Proteins No BLAST results-PFAM: Sjogren's syndrome/scleroderma autoantigen 1 (Autoantigen p27) | +2.29 |  |
| 12011 | *Cryptococcus neoformans var. neoformans B-3501A* | hypothetical protein CNBF4280 | +2.28 |  |
| 25162 | *Giardia lamblia ATCC 50803* | hypothetical protein GLP_0_6233_4263 | +2.27 |  |
| 24439 | *Gallus gallus* | PREDICTED: similar to HHM protein | +2.25 |  |
| 20795 | *4th Best Hit ACC No.: NP_012938 Saccharomyces cerevisiae* | 4th Best Hit:Protein of unknown function, has similarity to Pry1p and Pry3p and to the plant PR-1 class of pathogen related proteins; Pry2p | +2.24 |  |
| 5484 | *Schistosoma japonicum* | SJCHGC00927 protein | +2.23 |  |
| 11717 | *4th Best Hit ACC No.: YP_132408 Photobacterium profundum SS9* | 4th Best Hit:hypothetical protein PBPRB0736 | +2.22 |  |
| 5902 | *Xenopus laevis* | LOC494730 protein | +2.22 |  |
| 870 | - | Hypothetical Proteins No BLAST results-PFAM: MORN repeat | +2.17 |  |
| 23144 | *Canis familiaris* | PREDICTED: similar to dentin sialophosphoprotein preproprotein | +2.17 |  |
| 25355 | *Cryptosporidium parvum Iowa II* | putative Sec61 | +2.16 |  |
| 5105 | - | Hypothetical Protein No BLAST result | +2.16 |  |
| 35194 | *Gallus gallus* | PREDICTED: similar to Protein BAP28, partial | +2.13 |  |
| 1428 | - | Hypothetical Proteins No BLAST results-PFAM: Tubulin-tyrosine ligase family | +2.13 |  |
| 1069 | - | Hypothetical Proteins No BLAST results-PFAM: Protein of unknown function, DUF590 | +2.09 |  |
| 4160 | - | - | +2.08 |  |
| 5701 | - | Hypothetical Protein No BLAST result; PFAM: Thrombospondin type 1 domain | +2.07 |  |
| 7349 | *Vibrio splendidus 12B01* | putative site-specific recombinase, phage integrase family | +2.05 | (+3.25 pH)^b^ |
| 32037 | *Gallus gallus* | PREDICTED: similar to for proteasomal ATPase (SUG1) | +2.03 |  |
| 10774 | *Xenopus laevis* | MGC81025 protein | +2.00 |  |
| 9619 | *Aspergillus nidulans FGSC A4* | hypothetical protein AN7262.2 | -17.70 | (+6.95 Si)^b^ |
| 11430 | *-* | Hypothetical Protein No BLAST result | -6.00 |  |
| 9236 | *Dictyostelium minutum* | extracellular matrix protein B | -5.91 |  |
| 11059 | - | Hypothetical Protein No BLAST result; PFAM: HMG (high mobility group) box | -5.50 |  |
| 8898 | *-* | Hypothetical Protein No BLAST result | -4.96 |  |
| 3157 | - | Hypothetical Protein No BLAST result | -4.88 |  |
| 8111 | *Corynebacterium diphtheriae* | Putative membrane protein | -4.72 | (-5.6 Fe)^c^ |
| 7135 | - | Hypothetical Protein No BLAST result; PFAM: Glyoxalase/Bleomycin resistance protein/Dioxygenase superfamily | -4.71 |  |
| 4936 | *-* | Hypothetical Protein No BLAST result | -4.63 | (-5 Fe)^c^ |
| 11720 | - | Hypothetical Protein No BLAST result; PFAM: RNase | -4.36 |  |
| 2039 | *Xenopus laevis* | MGC53924: MGC53924 protein | -4.20 |  |
| 8281 | *-* | Hypothetical Protein No BLAST result | -4.17 | (+2.66 T)^b^ |
| 6750 | *-* | Hypothetical Protein No BLAST result | -4.13 |  |
| 23993 | *Tribolium castaneum* | PREDICTED: similar to CG10936-PA, isoform A | -4.12 |  |
| 6572 | *-* | Hypothetical Protein No BLAST result | -4.05 |  |
| 20837 | - | Hypothetical Protein No BLAST result; PFAM: Rhomboid family | -4.01 |  |
| 25088 | *-* | Hypothetical Protein No BLAST result | -3.89 | (-6.1 Fe)^c^ |
| 23623 | - | Hypothetical Protein No BLAST result; PFAM: SCP-like extracellular protein | -3.82 | (-2.08 Si; -1.79 Fe; +4.82 N)^b^ |
| 25749 | *-* | Hypothetical Protein No BLAST result | -3.81 |  |
| 23503 | *Saccharophagus degradans 2-40* | hypothetical protein Sde_3273 | -3.80 |  |
| 23562 | *Drosophila melanogaster* | CG15295-PA | -3.79 |  |
| 9314 | - | Hypothetical Protein No BLAST result; PFAM: UBA/TS-N domain | -3.77 |  |
| 9152 | *Dictyostelium discoideum* | hypothetical protein DDB0187034 | -3.72 | +2.8 (pH)^b^ |
| 22699 | *Staphylococcus epidermidis ATCC 12228* | streptococcal hemagglutinin protein | -3.69 |  |
| 7333 | *Leishmania major strain Friedlin* | proteophosphoglycan 5 | -3.67 |  |
| 37791 | *Rattus norvegicus* | LanC lantibiotic synthetase component C-like 2 | -3.67 |  |
| 23934 | *Plasmodium falciparum 3D7* | hypothetical protein, conserved | -3.60 |  |
| 3770 | *Arabidopsis thaliana* | AT1G27510: unknown protein | -3.59 |  |
| 7508 | *-* | Hypothetical Protein No BLAST result | -3.53 |  |
| 23108 | *Caenorhabditis elegans* | Hypothetical protein Y39B6A.1 | -3.52 |  |
| 8974 | *Leishmania major strain Friedlin* | proteophosphoglycan 5 | -3.52 |  |
| 11436 | *-* | Hypothetical Protein No BLAST result | -3.50 |  |
| 9406 | *Tribolium castaneum* | PREDICTED: similar to CCAAT/enhancer binding protein zeta | -3.50 |  |
| 12009 | *Dictyostelium discoideum* | hypothetical protein DDB0206366 | -3.49 |  |
| 21449 | *-* | Hypothetical Protein No BLAST result | -3.49 |  |
| 23947 | *-* | - | -3.47 |  |
| 25909 | *-* | Hypothetical Protein No BLAST result | -3.46 | (+2.06 pH)^b^ |
| 23521 | *-* | Hypothetical Protein No BLAST result | -3.35 |  |
| 7752 | *-* | Hypothetical Protein No BLAST result | -3.32 |  |
| 8895 | *-* | Hypothetical Protein No BLAST result | -3.30 |  |
| 25428 | *-* | - | -3.29 |  |
| 23825 | *Dictyostelium discoideum* | hypothetical protein DDB0203237 | -3.28 |  |
| 23948 | *-* | Hypothetical Protein No BLAST result | -3.28 |  |
| 11691 | *-* | Hypothetical Protein No BLAST result | -3.28 |  |
| 1949 | - | Hypothetical Proteins No BLAST results-PFAM: Isy1-like splicing family | -3.27 |  |
| 8782 | *-* | Hypothetical Protein No BLAST result | -3.24 |  |
| 10983 | - | Hypothetical Protein No BLAST result: ProSite- EF-hand calcium-binding domain | -3.19 | (+7.7 Fe)^c^ |
| 2928 | *Drosophila pseudoobscura* | Dpse\GA13585: GA13585-PA | -3.15 |  |
| 22669 | *-* | Hypothetical Protein No BLAST result | -3.13 |  |
| 23949 | *Leishmania major strain Friedlin* | proteophosphoglycan ppg4 | -3.11 |  |
| 25067 | *Homo sapiens* | dentin sialophosphoprotein preproprotein | -3.09 |  |
| 24595 | - | Hypothetical Protein No BLAST result; PFAM: Protein tyrosine kinase | -3.08 |  |
| 24870 | *Staphylococcus aureus subsp. aureus MSSA476* | putative cell wall-anchored protein | -3.07 |  |
| 21712 | *Arabidopsis thaliana* | unknown protein | -3.04 |  |
| 36929 | *3nd Hit: ACC No. NP_196063 Arabidopsis thaliana* | 3nd Hit:nucleic acid binding | -3.04 |  |
| 21076 | - | Hypothetical Protein No BLAST result; PFAM: Glycosyltransferase sugar-binding region containing DXD motif | -3.03 | (-2.21 Fe; +5.2 N)^b^ |
| 32693 | *Tribolium castaneum* | PREDICTED: similar to CG6375-PB, isoform B | -3.03 |  |
| 25467 | - | Hypothetical Protein No BLAST result; PFAM: Protein of unknown function (DUF672) | -3.03 |  |
| 6731 | - | Hypothetical Protein No BLAST result; PFAM: Domain of unknown function (DUF755) | -3.01 | (-3.23 N; -2.64 T)^b^ |
| 25825 | *-* | - | -3.01 |  |
| 7815 | *Actinomyces viscosus* | nanH: sialidase | -3.01 |  |
| 12025 | *Reinekea sp. MED297* | putative lipopolysaccharide A protein | -2.98 |  |
| 9393 | *-* | Hypothetical Protein No BLAST result | -2.97 |  |
| 7950 | *Dictyostelium discoideum* | hypothetical protein DDB0206375 | -2.95 |  |
| 1927 | *Staphylococcus epidermidis ATCC 12228* | SE2249: streptococcal hemagglutinin protein | -2.94 |  |
| 25594 | *Vibrio fischeri ES114* | chitodextrinase precursor | -2.93 |  |
| 23888 | *Leishmania major strain Friedlin* | proteophosphoglycan 5 | -2.92 |  |
| 6097 | *-* | Hypothetical Protein No BLAST result | -2.91 |  |
| 3037 | - | Hypothetical Proteins No BLAST results-PFAM: TolA protein | -2.87 |  |
| 25429 | *-* | - | -2.84 |  |
| 9162 | *Rhodopirellula baltica SH 1* | hypothetical protein-signal peptide prediction | -2.83 |  |
| 21732 | *4th Best Hit ACC No.: NP_001015825 Xenopus tropicalis* | 4th Best Hit:MGC97716 protein | -2.81 |  |
| 22481 | *Xenopus laevis* | B52-prov protein | -2.80 |  |
| 8732 | *4th Best Hit ACC No.: XP_647415 Dictyostelium discoideum* | 4th Best Hit:hypothetical protein DDB0189645 | -2.77 |  |
| 21438 | *Geobacter uraniumreducens Rf4* | conserved hypothetical protein | -2.76 |  |
| 23881 | *Homo sapiens* | dentin sialophosphoprotein precursor | -2.75 |  |
| 6273 | *Parachlamydia sp. UWE25* | conserved hypothetical protein | -2.75 |  |
| 2003 | - | Hypothetical Proteins No BLAST results-Prosite: Ubiquitin domain profile, PFAM: XPA protein C-terminus | -2.75 |  |
| 25181 | *Spodoptera litura nucleopolyhedrovirus* | essential structural protein pp78/81 | -2.75 |  |
| 269696 | *Thalassiosira pseudonana* | 90% identity with Cue4/Cue5 | -2.74 | (-4.53 Si, -3.62 Fe, -5.46 N, +3.03 pH)^b^; (Cu)^d^ |
| 23314 | *Navicula pelliculosa* | epsilon frustilin | -2.73 |  |
| 5757 | *Tribolium castaneum* | PREDICTED: similar to CG16733-PA | -2.73 |  |
| 21983 | *Caenorhabditis briggsae* | Hypothetical protein CBG23547 | -2.72 |  |
| 7282 | - | Hypothetical Protein No BLAST result: ProSite- EF-hand calcium-binding domain | -2.72 |  |
| 25335 | - | - | -2.70 |  |
| 262704 | *Thiobacillus denitrificans ATCC 25259* | hypothetical protein Tbd_0747 | -2.70 |  |
| 24571 | *-* | - | -2.70 | (-1.97 Fe)^b^ |
| 24011 | *Entamoeba invadens* | Jacob 6 | -2.68 |  |
| 25430 | *Leishmania major strain Friedlin* | proteophosphoglycan ppg4 | -2.67 |  |
| 23935 | *Candida albicans SC5314* | hypothetical protein CaO19_4906 | -2.66 |  |
| 2132 | *Caenorhabditis briggsae* | CBG21002: Hypothetical protein CBG21002 | -2.65 |  |
| 7709 | *Tribolium castaneum* | PREDICTED: similar to Ankyrin-1 (Erythrocyte ankyrin) (Ankyrin R) | -2.65 | (-3.68 Si; +3.15 N; -3.82 T)^b^ |
| 23396 | *Giardia lamblia ATCC 50803* | hypothetical protein GLP_516_15721_12530 | -2.64 |  |
| 264804 | *Acidobacteria bacterium Ellin345* | hypothetical protein Acid345_1066 | -2.62 |  |
| 25439 | *Leishmania major strain Friedlin* | proteophosphoglycan ppg4 | -2.61 | (-2.96 Si; -2.43 Fe)^b^ |
| 12149 | *Leishmania major strain Friedlin* | proteophosphoglycan ppg4 | -2.61 |  |
| 16607 | *3nd Hit: ACC No. NP_193076 Arabidopsis thaliana* | 3nd Hit:unknown protein | -2.61 |  |
| 24144 | *4th Best Hit ACC No.: AAY29120 Phragmatopoma californica* | 4th Best Hit:cement precursor protein 3B variant 1 | -2.59 |  |
| 262032 | *Canis familiaris* | PREDICTED: similar to Periaxin | -2.58 | (+2.3 Fe)^c^ |
| 24325 | *Nostoc sp. PCC 7120* | all1696 | -2.57 | (-3.38 T)^b^ |
| 22670 | *Arabidopsis thaliana* | unknown protein | -2.56 |  |
| 5605 | *3nd Hit: ACC No.* | 3nd Hit: | -2.55 |  |
| 18775 | *Schistosoma japonicum* | SJCHGC09106 protein | -2.55 |  |
| 25015 | *4th Best Hit ACC No.: XP_702405 Danio rerio* | 4th Best Hit:PREDICTED: hypothetical protein XP_697313 | -2.54 |  |
| 9145 | *Dictyostelium discoideum* | hypothetical protein DDB0205514 | -2.54 |  |
| 11897 | *uncultured bacterium 443* | twin-arginine translocation domain protein | -2.53 |  |
| 24748 | *-* | Hypothetical Protein No BLAST result | -2.52 |  |
| 24559 | - | Hypothetical Protein No BLAST result; PFAM: Thioredoxin | -2.52 |  |
| 25816 | *Eremothecium gossypii* | ABR064Wp | -2.52 |  |
| 21237 | *Methanosarcina barkeri str. fusaro* | conserved hypothetical protein | -2.51 |  |
| 10542 | *Dictyostelium discoideum* | hypothetical protein DDB0205674 | -2.50 |  |
| 6714 | - | Hypothetical Protein No BLAST result; PFAM: Protein of unknown function (DUF1501) | -2.48 |  |
| 23933 | *Thalassiosira pseudonana* | 25% identity with a copper-induced girdle band-associated cell surface protein precursor p150 | -2.47 | (Cu)^d^ |
| 24564 | *-* | - | -2.47 | (-2.68 Si; -2.99 Fe)^b^ |
| 9240 | *-* | - | -2.46 | (-2.84 Si; -2.88 Fe)^b^ |
| 24961 | *4th Best Hit ACC No.: XP_787093 Strongylocentrotus purpuratus* | 4th Best Hit:PREDICTED: similar to kelch-like 20 | -2.45 |  |
| 20974 | *Entamoeba invadens* | Jacob 6 | -2.44 |  |
| 262855 | *Pan troglodytes* | PREDICTED: similar to dJ132F21.2 (Contains a novel protein similar to the L82E from Drosophila) | -2.44 |  |
| 3006 | *Homo sapiens* | DSPP: dentin phosphoprotein | -2.43 |  |
| 6625 | *Colwellia psychrerythraea 34H* | hypothetical protein CPS_2065 | -2.42 | (-6.7 Fe)^c^ |
| 23794 | *Simian adenovirus 1* | pol | -2.42 | (-1.99 T)^b^ |
| 10539 | - | Hypothetical Protein No BLAST result; PFAM: Aldehyde dehydrogenase family | -2.38 |  |
| 21433 | *Drosophila melanogaster* | CG6004-PB | -2.36 |  |
| 6348 | *Arabidopsis thaliana* | unknown protein | -2.36 |  |
| 22003 | *3nd Hit: ACC No. NP_002448 Homo sapiens* | 3nd Hit:mucin 2 | -2.35 |  |
| 21400 | *Homo sapiens* | dentin sialophosphoprotein preproprotein | -2.35 |  |
| 10428 | *Candida albicans SC5314* | hypothetical protein CaJ7.0381 | -2.34 |  |
| 25395 | *Saccharomyces cerevisiae* | Protein involved in 5.8S rRNA processing; Ccr4p-like RNase required for correct 3'-end formation of 5.8S rRNA at site E; similar to Ngl1p and Ngl3p; Ngl2p | -2.34 |  |
| 264357 | *Arabidopsis thaliana* | AT5g44790/K23L20_14 | -2.34 |  |
| 10374 | *Schizosaccharomyces pombe 972h-* | hypothetical protein SPBC1711.05 | -2.33 |  |
| 9268 | *-* | Hypothetical Protein No BLAST result | -2.33 | (-2.4 T)^b^ |
| 23962 | - | Hypothetical Protein No BLAST result; PFAM: Atrophin-1 family | -2.33 |  |
| 24591 | *-* | Hypothetical Protein No BLAST result | -2.33 |  |
| 21861 | *Leishmania major strain Friedlin* | proteophosphoglycan ppg4 | -2.31 |  |
| 25820 | *Staphylococcus epidermidis RP62A* | serine threonine rich antigen | -2.31 |  |
| 24113 | *4th Best Hit ACC No.: ZP_01140530 Geobacter uraniumreducens Rf4* | 4th Best Hit:Sel1-like repeat | -2.31 |  |
| 25553 | - | Hypothetical Protein No BLAST result: ProSite- EF-hand calcium-binding domain | -2.30 |  |
| 262535 | *-* | - | -2.29 |  |
| 2612 | *Trypanosoma cruzi strain CL Brener* | Tc00.1047053506495.40: hypothetical protein | -2.29 |  |
| 9555 | *Vibrio splendidus 12B01* | Endonuclease I | -2.29 |  |
| 24029 | *Staphylococcus aureus* | serine-threonine rich antigen | -2.29 |  |
| 2015 | *Dictyostelium discoideum AX4* | DDBDRAFT_0167791: hypothetical protein | -2.28 | (+2.2 Fe)^c^ |
| 27836 | *Saccharomyces cerevisiae* | CSD2: chitin synthase | -2.26 |  |
| 2735 | *Dictyostelium discoideum AX4* | DDBDRAFT_0190016: hypothetical protein | -2.25 |  |
| 21808 | *Rhodopirellula baltica SH 1* | hypothetical protein-signal peptide prediction | -2.24 |  |
| 9657 | *-* | Hypothetical Protein No BLAST result | -2.23 |  |
| 23895 | *-* | - | -2.22 |  |
| 2562 | - | Hypothetical Proteins No BLAST results-Prosite: Type-1 copper (blue) proteins signature, PFAM: Exostosin family | -2.21 |  |
| 9031 | *Dictyostelium discoideum* | hypothetical protein DDB0186436 | -2.20 |  |
| 22002 | *Toxocara canis* | excretory/secretory mucin MUC-3 | -2.19 |  |
| 23894 | *Leishmania major strain Friedlin* | proteophosphoglycan 5 | -2.18 |  |
| 22748 | *Wolbachia endosymbiont of Drosophila ananassae* | ankyrin repeat domain protein | -2.17 |  |
| 10279 | *Xenopus laevis* | LOC496006 protein | -2.17 |  |
| 23725 | *Tribolium castaneum* | PREDICTED: similar to CG32732-PA | -2.16 |  |
| 34043 | *Caenorhabditis elegans* | Hypothetical protein ZK909.2h | -2.15 |  |
| 25867 | *Drosophila melanogaster* | CG1814-PA, isoform A | -2.15 |  |
| 6292 | *Vibrio fischeri ES114* | chitodextrinase precursor | -2.14 |  |
| 11029 | *Homo sapiens* | KIAA0324 protein | -2.13 |  |
| 6715 | *3nd Hit: ACC No. ZP_01218461 Photobacterium profundum 3TCK* | 3nd Hit:hypothetical protein P3TCK_20840 | -2.13 |  |
| 263298 | *Ustilago maydis* | class V chitin synthase | -2.11 |  |
| 263301 | *Ustilago maydis* | class V chitin synthase | -2.10 |  |
| 24149 | Hypothetical Proteins No BLAST results-PFAM: Zinc finger, C3HC4 type (RING finger) 35973kDa | Hypothetical Protein No BLAST result | -2.09 |  |
| 21809 | *3nd Hit: ACC No. NP_828796 Streptomyces avermitilis MA-4680* | 3nd Hit:putative ATP/GTP-binding Gly/Ala-rich protein | -2.09 |  |
| 10749 | *-* | - | -2.08 |  |
| 269515 | *Debaryomyces hansenii CBS767* | hypothetical protein DEHA0F15235g | -2.08 |  |
| 23899 | *Debaryomyces hansenii CBS767* | hypothetical protein DEHA0G26499g | -2.07 |  |
| 25591 | *Vibrio vulnificus CMCP6* | Uncharacterized protein contain chitin-binding domain type 3 | -2.07 |  |
| 4736 | *-* | Hypothetical Protein No BLAST result | -2.05 |  |
| 22971 | - | Hypothetical Protein No BLAST result; PFAM: Cytochrome b5-like Heme/Steroid binding domain | -2.05 |  |
| 22709 | *Strongylocentrotus purpuratus* | PREDICTED: hypothetical protein XP_795427, partial | -2.04 |  |
| 24002 | *-* | Hypothetical Protein No BLAST result | -2.04 |  |
| 24991 | *Dictyostelium discoideum* | argonaut-like protein | -2.04 |  |
| 24926 | *Mus musculus* | PREDICTED: similar to TD and POZ domain containing 5 | -2.03 |  |
| 22128 | *Xenopus laevis* | LOC446246 protein | -2.03 |  |
| 24067 | *Plasmodium berghei strain ANKA* | hypothetical protein PB000065.03.0 | -2.03 |  |
| 21390 | - | Hypothetical Protein No BLAST result | -2.02 |  |
| 20982 | *Anaeromyxobacter dehalogenans 2CP-C* | GPR1/FUN34/yaaH | -2.02 |  |
| 5548 | *-* | Hypothetical Protein No BLAST result | -2.00 |  |

^a^ Annotation on gene function was taken from (Nunn et al. 2009) using BLAST analysis against databases of other organisms.

^b^ Genes previously reported as being differentially expressed under silicon (Si)-, iron (Fe)-, nitrogen (N)-, or temperature (T)-limitation or alkaline (pH) relative to nutrient-replete growth (Mock et al. 2008)

^c^ Genes previously reported as being differentially expressed under low iron (Fe)-availability (Thamatrakoln et al. 2011)

^d^ Genes previously reported as being up-regulated under conditions of copper (Cu)-induced stress (Davis et al. 2006)
