# Supplementary material for: Transcriptomics Responses in Marine Diatom Thalassiosira pseudonana Exposed to the Polycyclic Aromatic Hydrocarbon Benzo[a]pyrene
Source: PLoS One. 2011 Nov 3;6(11):e26985. doi: 10.1371/journal.pone.0026985 (PMC3207822; doi:10.1371/journal.pone.0026985)
Supplement: Table S2 — GO biological processes that are regulated by BaP in the diatom T. pseudonana . (DOC) [file pone.0026985.s003.doc]

**Table S2. GO biological processes that are regulated by BaP in the diatom *T. pseudonana***

| **GO ID** | **GO Biological Process** | **# Genes regulated** | **# Genes on the array** |
| --- | --- | --- | --- |
| UPREGULATED |  |  |  |
| GO:0009306 | protein secretion | 2 | 7 |
| GO:0009401 | phosphoenolpyruvate-dependent sugar phosphotransferase system | 2 | 9 |
| GO:0030163 | protein catabolic process | 2 | 9 |
| GO:0006511 | ubiquitin-dependent protein catabolic process | 5 | 49 |
| GO:0006118 | electron transport | 30 | 300 |
| GO:0006810 | transport | 17 | 202 |
| GO:0006457 | protein folding | 7 | 97 |
| GO:0006508 | proteolysis | 21 | 286 |
| DOWNREGULATED |  |  |  |
| GO:0030154 | cell differentiation | 2 | 3 |
| GO:0006032 | chitin catabolic process | 3 | 8 |
| GO:0007155 | cell adhesion | 3 | 8 |
| GO:0006030 | chitin metabolic process | 5 | 22 |
| GO:0000074 | regulation of cell cycle | 6 | 55 |
| GO:0005975 | carbohydrate metabolic process | 6 | 55 |
| GO:0009664 | plant-type cell wall organization | 3 | 28 |
| GO:0015986 | ATP synthesis coupled proton transport | 4 | 36 |
| GO:0006355 | regulation of transcription, DNA-dependent | 25 | 299 |
